# Aqueous-based recycling of perovskite photovoltaics

**DOI:** 10.1038/s41586-024-08408-7

**Published:** 2025-02-12

**Authors:** Xun Xiao, Niansheng Xu, Xueyu Tian, Tiankai Zhang, Bingzheng Wang, Xiaoming Wang, Yeming Xian, Chunyuan Lu, Xiangyu Ou, Yanfa Yan, Licheng Sun, Fengqi You, Feng Gao

**Affiliations:** 1 Department of Physics, Chemistry and Biology, Linköping University, Linköping, Sweden; 2 Systems Engineering, College of Engineering, Cornell University, Ithaca, NY USA; 3 Department of Physics and Astronomy, The University of Toledo, Toledo, OH USA; 4 Wright Center for Photovoltaics Innovation and Commercialization, The University of Toledo, Toledo, OH USA; 5 Center of Artificial Photosynthesis for Solar Fuels, Westlake University, Hangzhou, China; 6 Department of Chemistry, School of Science, Westlake University, Hangzhou, China; 7 Research Center for Industries of the Future, Westlake University, Hangzhou, China; 8 Division of Solar Energy Conversion and Catalysis at Westlake University, Zhejiang Baima Lake Laboratory Co., Ltd, Hangzhou, China; 9 Robert Frederick Smith School of Chemical and Biomolecular Engineering, Cornell University, Ithaca, NY USA; 10 Cornell Atkinson Center for Sustainability, Cornell University, Ithaca, NY USA; 11 Wallenberg Initiative Materials Science for Sustainability, Department of Physics, Chemistry and Biology, Linköping University, Linköping, Sweden

**Keywords:** Materials for energy and catalysis, Renewable energy, Energy, Solar cells

## Abstract

Cumulative silicon photovoltaic (PV) waste highlights the importance of considering waste recycling before the commercialization of emerging PV technologies^1,2^. Perovskite PVs are a promising next-generation technology^3^, in which recycling their end-of-life waste can reduce the toxic waste and retain resources^4,5^. Here we report a low-cost, green-solvent-based holistic recycling strategy to restore all valuable components from perovskite PV waste. We develop an efficient aqueous-based perovskite recycling approach that can also rejuvenate degraded perovskites. We further extend the scope of recycling to charge-transport layers, substrates, cover glasses and metal electrodes. After repeated degradation–recycling processes, the recycled devices show similar efficiency and stability compared with the fresh devices. Our holistic recycling strategy reduces by 96.6% resource depletion and by 68.8% human toxicity (cancer effects) impacts associated with perovskite PVs compared with the landfill treatment. With recycling, the levelized cost of electricity also decreases for both utility-scale and residential systems. This study highlights unique opportunities of perovskite PVs for holistic recycling and paves the way for a sustainable perovskite solar economy.

## Abstract (web summary)

A recycling strategy based predominantly on the use of water to restore and reuse valuable components from perovskite photovoltaic waste is described, with recycled devices showing similar efficiency and stability compared with fresh devices.

## Main

PVs provide a rapidly growing market to offer sustainable and low-cost electricity^6,7^. Nevertheless, the surging growth of PV technology leads to the accumulation of end-of-life PV modules, posing a growing demand for effective PV waste management^1,8^. Recycling these waste modules to recover valuable raw materials and facilitate the creation of new modules emerges as an economically viable and ecologically sound solution^2,9^. Therefore, nations worldwide are imposing extended producer responsibility on PV manufacturers, making producers responsible for collecting and recycling their PV product waste, such as WEEE directive 2012/19/EU in the European Union and equivalent legislation in Asia and the United States^10–13^.

Emerging perovskite PVs are promising for commercialization, with their advances in high power conversion efficiency and low cost^3^. With lessons learnt from the evolution of silicon PVs, it has become a prerequisite to develop recycling technologies for a sustainable PV system before their widespread adoption^5,14^. This is of particular importance for perovskite PVs, as it can also mitigate lead consumption and effectively manage the toxic lead-containing waste associated with perovskite solar modules^4,15,16^. Typical recycling approaches in perovskite PVs include layer-by-layer dissolution with solvents, such as dimethylformamide (DMF), chlorobenzene or methylamine, followed by extraction/redeposition of the functional materials^17–21^ (Supplementary Note 1). Although the recycling efficiency is optimized for these recycling strategies, the reliance on hazardous solvents brings substantial environmental concerns and compatibility issues with industrial processes^22–26^.

Here we report a green-solvent-based holistic recycling strategy towards the sustainable development of perovskite PVs. We successfully restore almost all of the essential functional materials, including hole/electron transport layers, the perovskite layer, indium tin oxide (ITO) substrates and cover glasses, with high recycling efficiency and purity. Our aqueous-based perovskite recycling approach, in which we immerse degraded perovskites into an aqueous solution for repairing and reclaiming high-quality perovskite crystals, has achieved an impressive 99.0 ± 0.4 wt% recycling efficiency. We demonstrate that our recycling strategy can greatly alleviate the environmental burden of waste perovskite modules, especially for the human toxicity and resource depletion impacts, and also reduce the levelized cost of electricity. This study demonstrates unique opportunities of holistic perovskite solar module recycling—which are barely possible for other PV technologies—for a sustainable and circular solar economy in the future.

## Circular perovskite solar economy

**Extended Data Fig. 1:**
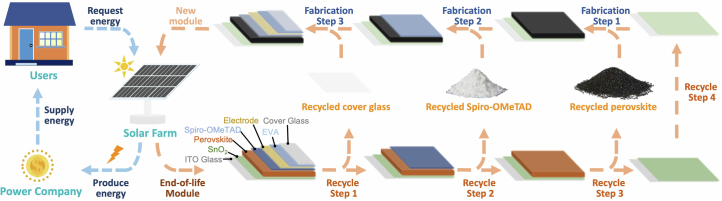
Scheme of proposed sustainable perovskite PVs with holistic recycling. The solar farm produces and supplies energy to support society. The end-of-life solar modules from the solar farm are repaired and recycled layer by layer. The recycled materials are then used to fabricate new solar modules and build new solar farms.

We propose a perovskite PV system that integrates energy generation with solar module recycling (Extended Data Fig. 1). In this system, perovskite solar farms continuously generate sustainable and low-cost electricity to fulfil societal energy demands. Once solar modules reach the end of their lifespan, they are recycled to fabricate new perovskite PV modules. In the recycling process, we start with thermal treatment of the waste modules at 150 °C for 3 min to soften the ethylene vinyl acetate (EVA) encapsulant, facilitating delamination (Supplementary Fig. 1). The delaminated modules are then layer-by-layer recycled to reclaim cover glass, spiro-OMeTAD, perovskite crystal powders and SnO_2_-coated ITO substrates. These recycled materials are subsequently used in the fabrication of new solar modules, thereby completing a full circular loop for perovskite PVs.

## Aqueous solution for perovskite recycling

**Fig. 1:**
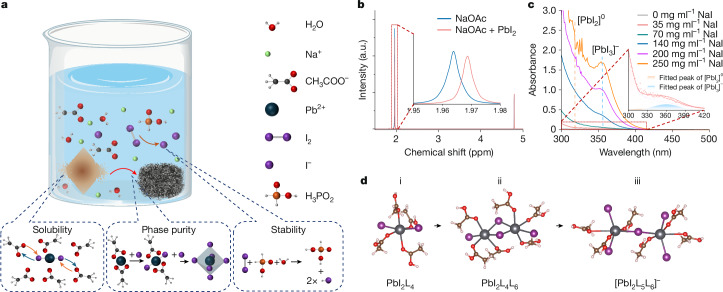
An aqueous-based solution for perovskite recycling. **a**, Scheme of recycling process with a water-based solution. Three main additives (NaOAc, NaI and H_3_PO_2_) are added to address perovskite solubility, phase purity and solution stability issues in water solution. **b**, ^1^H-NMR of NaOAc with and without PbI_2_. The chemical shift suggests a strong interaction between acetate and lead ions to facilitate the PbI_2_ dissolution in water. **c**, Absorption curves of water-based solution (NaOAc: 500 mg ml^−1^, FAPbI_3_: 1 μmol) with various NaI concentrations. The inset shows solution with 35 mg ml^−1^ NaI fitted with [PbI_2_]^0^ and [PbI_3_]^−^ peaks. **d**, DFT calculation of acetate ions coordination behaviour for facilitating lead iodide dissolution and phase change with the addition of iodide ions in water solution for perovskite recycling. Structures i, ii and iii denote PbI_2_L_4_, Pb_2_I_4_L_6_ and [Pb_2_I_5_L_6_]^−^, respectively, in which L is the acetate ions. a.u., arbitrary units. Source Data

**Extended Data Fig. 2:**
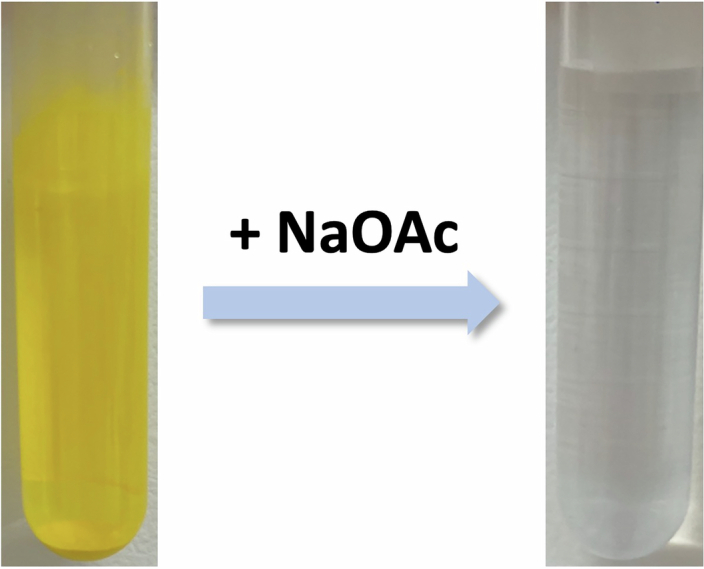
The role of acetate ions in dissolving perovskites in aqueous solution. Dissolving 100 mg of MAPbI_3_ powder in pure water (left) and adding sodium acetate (500 mg ml^−1^) to assist in dissolving PbI_2_ (right).

The recycling of the lead-containing perovskite layer constitutes a critical aspect of the sustainable perovskite PV development. We have developed an eco-friendly aqueous solution approach for perovskite recycling (Fig. 1a). Within this solution, we introduce three low-cost additives, sodium acetate (NaOAc), sodium iodide (NaI) and hypophosphorous acid (H_3_PO_2_), to address solubility, phase purity and stability challenges in the aqueous-based environment. Notably, water exhibits limited solubility towards lead iodide (about 0.044 g per 100 ml at 20 °C)^27^, despite its ability to dissolve organic iodide salts such as methylammonium iodide and formamidinium iodide. To enhance lead iodide dissolution in water, we introduce acetate ions that readily coordinate with lead ions, forming highly soluble lead acetate (about 44.31 g per 100 ml at 20 °C)^28^. This coordinative effect is evident by comparing the ^1^H-NMR spectrum of sodium acetate with and without added lead iodide (Fig. 1b), in which a distinct chemical shift in the acetate group indicates their strong interaction with lead ions. We make use of methylammonium lead iodide (MAPbI_3_) to visualize the efficacy of acetate ions in enhancing perovskite dissolution, to which we add 100 mg of MAPbI_3_ to 4 ml of pure water (Extended Data Fig. 2). After 10 min, undissolved yellow powder remains, indicating the limited solubility of lead iodide in water. Subsequently, with the introduction of a 500 mg ml^−1^ sodium acetate into solution, the lead iodide powder dissolves within 10 s of shaking. These results indicate that acetate ions can effectively facilitate perovskite dissolution in water through strong coordination with PbI_2_.

**Extended Data Fig. 3:**
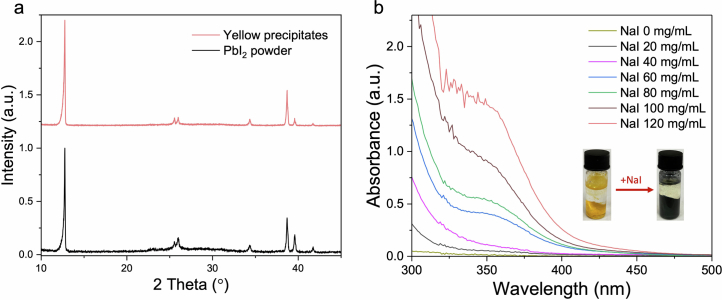
The role of NaOAc and NaI in the aqueous solution. **a**, XRD of PbI_2_ and precipitated yellow crystals after dissolving perovskite in NaOAc aqueous solution. **b**, Absorption curves of water-based solution (NaOAc: 500 mg ml^−1^; MAPbI_3_: 10 μmol) with various NaI concentrations. The emerging peak at about 360 nm with increasing NaI concentration is ascribed to the formation of [PbI_3_]^−^. The inset images help visualize the lead coordination variation and corresponding precipitate changes, in which we introduce an excessive amount (400 mg ml^−1^) of MAPbI_3_ into the NaOAc aqueous solution and then add NaI. The yellow powder of PbI_2_ could be transformed into the black perovskite phase of MAPbI_3_ following the addition of NaI. Source Data

With degraded perovskites dissolved in the aqueous solution, the next challenge is to recover phase-pure and high-quality perovskite crystals from the solution^27^. We initially use the temperature-dependent solubility in the NaOAc aqueous solution to recover the perovskite crystals, in which we dissolve the degraded MAPbI_3_ to saturation at high temperature and then cool it down to precipitate. However, this process yields yellow crystals with X-ray diffraction (XRD) pattern aligning well with PbI_2_ (Extended Data Fig. 3a). The results can be attributed to the formation of face-sharing or edge-sharing phases of [PbI]^+^ or [PbI_2_]^0^ surrounded by acetate ions^29^, rather than [PbI_3_]^−^ required for perovskite crystals. We notice that the phase of lead species in the solution can be precisely tuned by carefully manipulating the coordinative ions^29^. We thus gradually substitute acetate anions with iodide ions to coordinate with lead, successfully resulting in the transition from [PbI]^+^ to [PbI_2_]^0^ and eventually to [PbI_3_]^−^ for the formation of the perovskite framework. In this case, although acetate ions partially retain their coordination with the lead ions for sufficient solubility, the addition of iodide ions plays a crucial role in the phase control. The whole process is monitored by measuring the absorption of perovskites in NaOAc aqueous solution (Fig. 1c and Extended Data Fig. 3b) with an increasing amount of NaI for supplying iodide ions. Although the [PbI]^+^ peak located at about 290 nm is beyond the measurable range of our instrument, the transition to the dominant [PbI_3_]^−^ peak at approximately 360 nm is clearly identified by increasing the iodide concentration^29^. We visualize the phase transition from the yellow PbI_2_ powder to black MAPbI_3_ crystals with iodine addition in the aqueous solution (inset of Extended Data Fig. 3b), highlighting the critical role of iodide ions in tuning the phase purity of precipitates. We further perform density functional theory (DFT) calculations to reveal the mechanisms of ion-assisted phase changes in the aqueous solution (Fig. 1d), for which we find that the iodide ions assist the octahedral phase change from edge-sharing to corner-sharing configuration (Supplementary Note 2).

**Extended Data Fig. 4:**
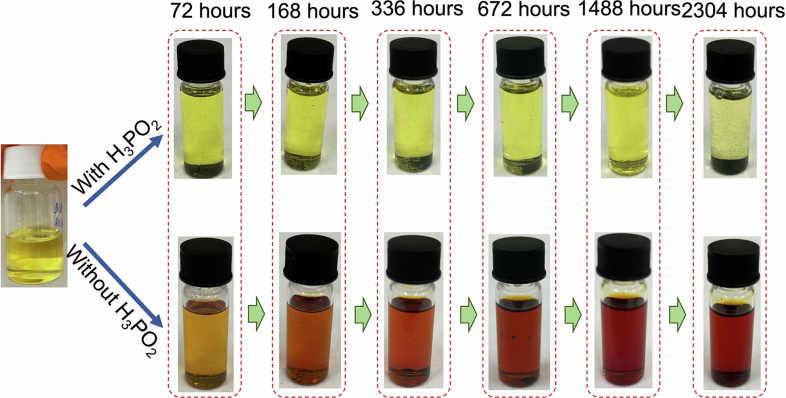
Thermal stability test for recycling solution. Aqueous-based recycling solutions with or without H_3_PO_2_ stabilizer were placed at a temperature of 95 °C. The yellow-to-brown colour change indicates iodine formation in solution without H_3_PO_2_ stabilizer. The images were taken by moving solutions from the hotplate to a flat table for a clear background. The amount of perovskite powder precipitates varies in different images because the solution temperature may be different as cooling occurs while taking pictures.

The third additive of H_3_PO_2_ is introduced as a stabilizer to ensure the long-term and high-temperature stability and reusability of the solution. In solutions with high iodide ion concentrations, iodide ions can be rapidly oxidized to iodine (I_2_) by atmosphere oxygen, particularly under higher temperatures. This oxidation can greatly reduce the iodide concentration, resulting in ineffective iodide-ions-assisted phase changes. Hence, we incorporate H_3_PO_2_ to reduce iodine to iodide ions through the reaction H_3_PO_2_ + I_2_ + H_2_O → H_3_PO_3_ + 2HI (ref. ^30^). As demonstrated in Extended Data Fig. 4, we divide the solution (aqueous solution of perovskites with NaI and NaOAc as additives) into two bottles and then add H_3_PO_2_ to one of them. Both solutions undergo a thermal stress test at 95 °C. The solution containing H_3_PO_2_ shows no colour change even after enduring more than 2,000 h of thermal stress and can still produce high-quality perovskite crystals. By contrast, the solution without H_3_PO_2_ exhibits a quick shift to brown colour within the first 72 h, progressing to dark red with extended treatment time, indicating rapid iodine formation. The substantial improvement in solution stability with the H_3_PO_2_ additive makes aqueous-based recycling solution reusable.

Equipped with the additives, the aqueous solution is optimized to feature enhanced perovskite solubility, phase-pure crystal growth capability and excellent thermal stability. Therefore, we can achieve a waste-free and almost 100% atomic efficiency recycling through the temperature-dependent solubility of perovskites in the aqueous solution. Specifically, we immerse the degraded perovskite film into the hot aqueous solution (around 80 °C), in which the increased solubility at high temperature facilitates the dissolution of perovskites. We then remove the substrate and gradually cool the solution to precipitate high-purity perovskite crystals for waste-free recycling (Supplementary Video 1), for which we use MAPbI_3_ as an example. The XRD peaks of the recycled powder and films (prepared from recycled powders) align well with phase-pure MAPbI_3_ (Supplementary Fig. 2). Perovskite recycling efficiency is assessed by three independent recycling iterations, yielding an average recycling efficiency of 99.0 ± 0.4 wt%.

**Fig. 2:**
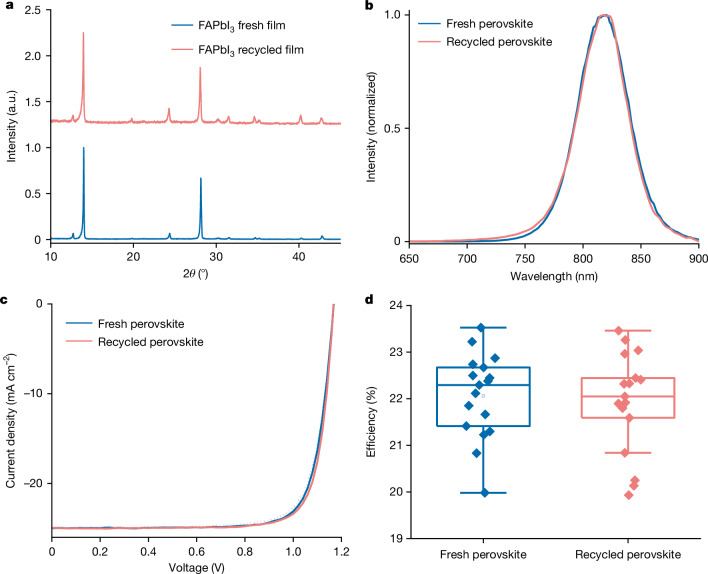
Perovskite recycling. **a**–**d**, XRD pattern (**a**) and photoluminescence spectra (**b**) of FAPbI_3_ films with fresh and recycled perovskite materials. *J*–*V* curves (**c**) and statistical efficiency chart (**d**) of devices with fresh and recycled FAPbI_3_ perovskite, in which 17 devices are summarized in the box plot of **d**. a.u., arbitrary units. Source Data

**Extended Data Fig. 5:**
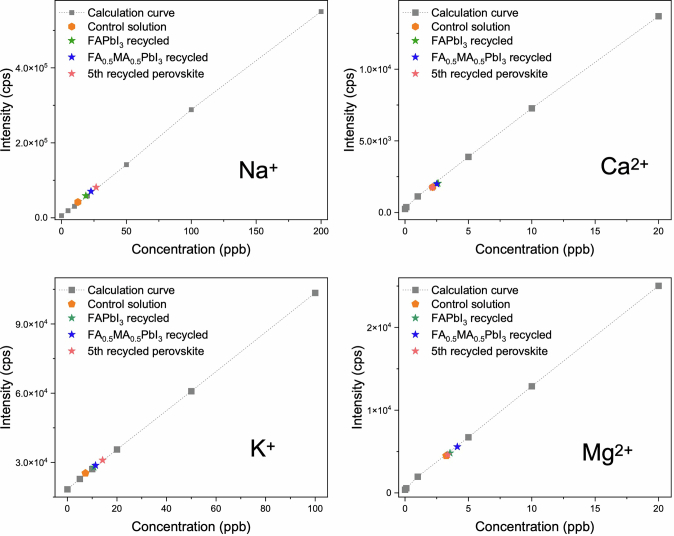
ICP-MS measurement of different impurity elements in recycled perovskites. Certain amounts (FAPbI_3_ recycled: 0.895 mg; FA_0.5_MA_0.5_PbI_3_ recycled: 1.040 mg; fifth-round recycled perovskite: 0.930 mg) of recycled perovskite powder are dissolved in the plastic tube with a 3 ml mixture of nitric acid, hydrochloride acid and pure water (1:1:1). The solution is then diluted by 20 times with pure water for the ICP-MS measurement. The calibration curves and the measured diluted samples are averaged from three samplings for each test. A control solution with an identical amount of pure solvents is measured for reference. The mass fractions of impurities are determined by subtracting the solvent control and considering the diluting effect during measurement. The purity (*P*) of our recycled perovskite crystal is calculated as $P( \% )=\left(1-{\sum }_{i}^{n}{W}_{{\rm{i}}}\right)\times 100 \%$, in which *W*_i_ is the mass fraction of impure elements and *n* is the number of impure elements. Source Data

We also apply this technology to recycle FAPbI_3_ perovskites. The thin film produced by recycled crystals exhibits nearly identical XRD patterns, photoluminescence spectra and morphology to that made from fresh materials (Fig. 2a,b and Supplementary Fig. 3). We conducted inductively coupled plasma mass spectrometry (ICP-MS) to examine the purity of recycled perovskite, which reaches 99.999312% with dominant sodium impurity of 4.1 ppm (Extended Data Fig. 5). The devices made with recycled perovskites achieve an average power conversion efficiency of 21.9 ± 1.1%, with a champion value of 23.4%. It represents an efficiency recovery of more than 99% compared with those prepared with fresh materials (22.1 ± 0.9%) (Fig. 2c,d), benefiting from the high-quality crystal growth during recycling. We also demonstrate the generalized applicability to recycle mixed-cation perovskites (FA_0.5_MA_0.5_PbI_3_ as an example), resulting in the negligible difference in XRD patterns and purity (Supplementary Fig. 4). Also, our recycling process can repair degraded perovskites (Supplementary Fig. 5 and Supplementary Note 3).

## Holistic and multi-round recycling

**Fig. 3:**
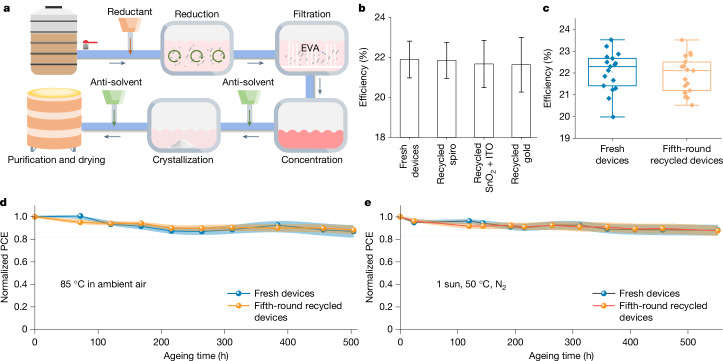
Holistic and multi-round recycling. **a**, Scheme of recycling process of spiro-OMeTAD, in which the reductant is iodide salt, dissolving solvent is EA and anti-solvent is ethanol. **b**, Statistical device efficiency results with different recycled components. **c**, Statistical efficiency chart of fresh and fifth-round recycled devices. Different from specific recycled components, recycled devices mean that all of the materials (including perovskite, spiro, SnO_2_ + ITO substrate and gold) for device fabrication are recycled. **d**, Efficiency loss of fresh and fifth-round recycled devices stored in ambient air at 85 °C. **e**, Operational stability under one-sun light soaking at 50 °C in nitrogen. The error bar indicates the standard deviation from five independent measurements. All devices are unencapsulated and in the open-circuit condition. PCE, power conversion efficiency. Source Data

**Extended Data Fig. 6:**
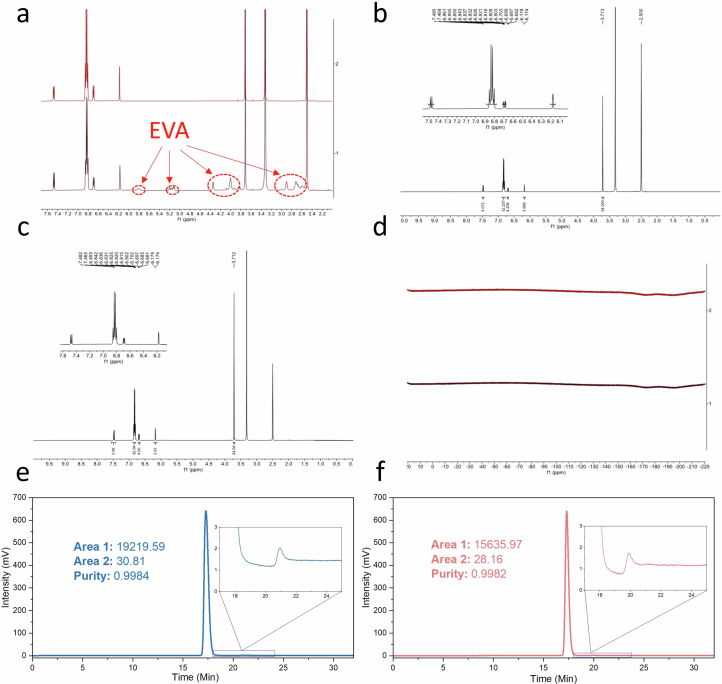
Characterization of recycled spiro-OMeTAD. **a**, ^1^H-NMR spectra of recycled spiro-OMeTAD in DMSO-*d*_6_ with (top) or without (bottom) filtration to remove EVA. ^1^H-NMR spectra of recycled spiro-OMeTAD (**b**) and fresh spiro-OMeTAD (**c**) in DMSO-*d*_6_. **d**, ^19^F-NMR spectra of fresh (top) and recycled (bottom) spiro-OMeTAD in DMSO-*d*_6_. High-performance liquid chromatography spectra of fresh (**e**) and recycled (**f**) spiro-OMeTAD. Source Data

**Extended Data Fig. 7:**
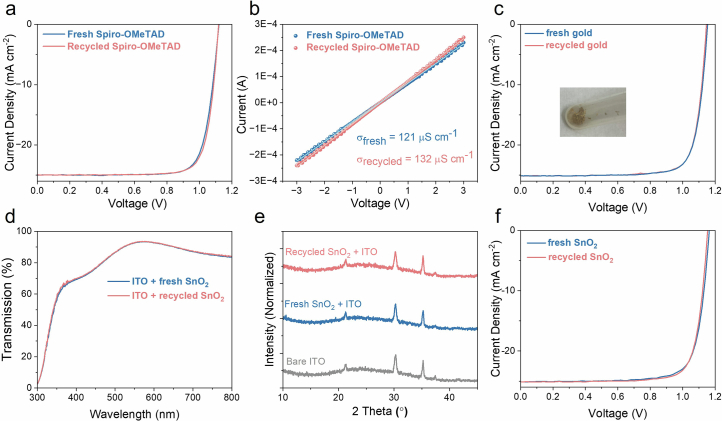
Recycling all valuable components. **a**, *J*–*V* curves of devices made with fresh and recycled spiro-OMeTAD. **b**, Conductivity of fresh and recycled spiro-OMeTAD doped with the same ion-modulated radical doping method. **c**, *J*–*V* curves of devices by fresh and recycled gold electrode, in which the inset is the recycled gold. **d**,**e**, Transmission and XRD patterns of recycled ITO glass with SnO_2_. **f**, *J*–*V* curves of devices on fresh and recycled SnO_2_-coated ITO glass. Source Data

To realize a holistic recycling for perovskite PVs, we further develop recycling approaches for all valuable components in waste perovskite solar modules. First, we develop a recycling method to recover the hole-transport material, spiro-OMeTAD, with green solvents, that is, ethyl acetate (EA) and ethanol (Fig. 3a and Supplementary Fig. 6). By eliminating impurities and reduction in the recycling process (Supplementary Note 4), the purity of recycled neutral spiro-OMeTAD is measured with high-performance liquid chromatography to be 99.82%, which is close to that of fresh material of 99.84% (Extended Data Fig. 6). The recycling efficiency is assessed by three independent cycles to be 97.8 ± 0.3 wt%. The recycled spiro-OMeTAD exhibits nearly identical conductivity and device efficiency compared with those of fresh materials (Fig. 3b and Extended Data Fig. 7).

The gold electrode is recycled by centrifuging the EA solution of spiro-OMeTAD to collect the solid electrode. The solar cells made with fresh and recycled gold electrodes show negligible differences (Fig. 3b). Finally, the SnO_2_-coated ITO glasses are recycled through cleaning and ultraviolet–ozone treatment to remove the defects in SnO_2_ (ref. ^31^). The recycled substrates exhibit similar optical and crystalline properties and device performance as compared with the fresh ones (Extended Data Fig. 7).

We investigate the multi-round recycling capability of our holistic recycling strategy with a repeated degradation–recycling process (five rounds are demonstrated). Each degradation process is accelerated with 85 °C thermal stress and one-sun illumination under ambient conditions (relative humidity approximately 60%) until the unencapsulated devices reach greater than 20% efficiency loss. The fifth-round recycled devices achieve an average power conversion efficiency of 21.8 ± 0.8%, with a champion efficiency of 23.5% (Fig. 3c and Supplementary Figs. 7 and 8), comparable with those of the devices fabricated with fresh materials. Note that the aqueous solution after multi-round recycling of perovskites exhibits negligible composition change, consistently producing high-quality perovskites crystals with a purity of 99.998644% (Supplementary Fig. 9).

To further assess the reliability of the recycled devices (unencapsulated), we conduct stability tests under two scenarios: (1) stored in ambient air at 85 °C and (2) one-sun light soaking at 50 °C in nitrogen. The results demonstrate that the fifth-round recycled devices retain 88.2 ± 4.0% of their initial efficiency after being stored for 504 h in ambient air at 85 °C and 87.7 ± 4.6% with 552 h of light soaking at 50 °C (Fig. 3d,e), comparable with devices made from fresh materials.

## Environmental and economic analysis

**Fig. 4:**
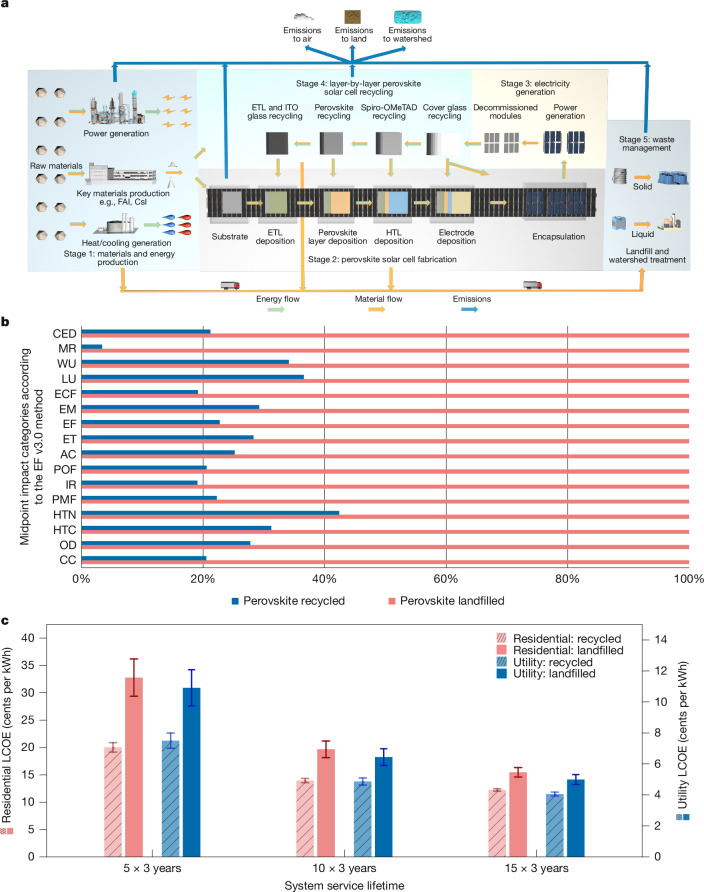
LCA and techno-economic analysis results. **a**, System boundary of LCA considering the proposed recycling strategy as the end-of-life scenario. **b**, Comparison of full-spectrum midpoint impact categories between recycling and landfill according to the Environmental Footprint (EF) v3.0 method (the values are normalized to the landfill scenario for better comparison). CED, cumulative energy demand; MR, material resources: metals/minerals; WU, water use; LU, land use; ECF, ecotoxicity: freshwater; EM, eutrophication: marine; EF, eutrophication: freshwater; ET, eutrophication: terrestrial; AC, acidification; POF, photochemical oxidant formation: human health; IR, ionizing radiation: human health; PMF, particulate matter formation; HTN, human toxicity: non-cancer effect; HTC: human toxicity: cancer effect; OD, ozone depletion; CC, climate change. **c**, LCOE of residential and utility-scale perovskite PV systems under recycling (three times) and landfill scenarios. Three device lifetimes are considered: 5 years, 10 years and 15 years. Therefore, the total service times are 5 × 3 and 10 × 3 and 15 × 3 years considering repeated recycling for the three scenarios to emphasize the importance of considering both lifespan and recycling times when assessing the economic efficiency of perovskite solar cells. The error bars convey the uncertainty in the LCOE estimates, reflecting a ±20% fluctuation in crucial input factors. This variability includes the expenses linked to energy consumption, labour, materials and equipment, all of which are integral to the processes of recycling, remanufacturing and reinstalling PV modules. ETL, electron transport layer; HTL, hole transport layer. Source Data

**Extended Data Fig. 8:**
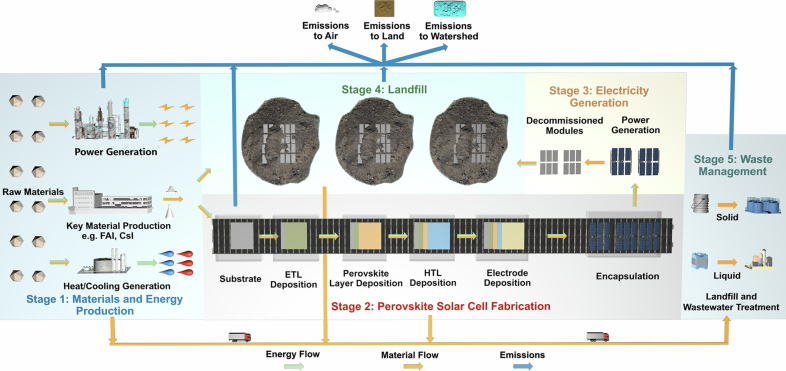
System boundary of manufacturing perovskite solar cell modules with landfill as the end-of-life scenarios. The system boundary includes the life-cycle stages of raw material acquisition, perovskite solar cell fabrication, electricity generation, landfills of end-of-life devices and waste management.

We carried out a comparative life-cycle assessment (LCA) to quantify the environmental benefits of the proposed recycling strategy by comparing it with landfill disposal as an end-of-life scenario for waste PV modules. The system boundaries are illustrated respectively in Fig. 4a for our recycling method and Extended Data Fig. 8 for the landfill scenario, in which we quantify the input of raw materials, energy consumption and emissions from the systems. We build a ‘cradle-to-grave’ system boundary for the landfill scenario, including the life-cycle stages of raw material acquisition, perovskite solar cell fabrication, electricity generation, landfills of end-of-life devices and waste management. By comparison, our recycling method is modelled in a ‘cradle-to-cradle’ system boundary (Fig. 4a), in which the end-of-life devices are dismantled and key components are recycled layer by layer to remanufacture new devices. The life-cycle stages of raw material acquisition, perovskite solar cell fabrication, electricity generation and waste management are the same across both scenarios.

**Extended Data Fig. 9:**
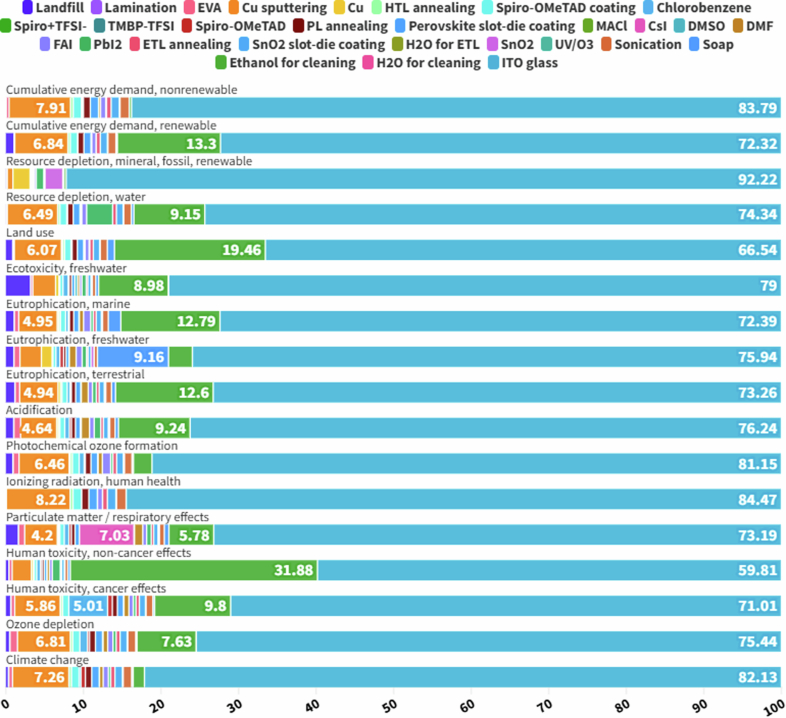
Contribution analysis results for the landfilled scenario. The perovskite solar cell with a ‘cradle-to-grave’ system boundary is considered. The figure is generated using the Flourish online tool.

**Extended Data Fig. 10:**
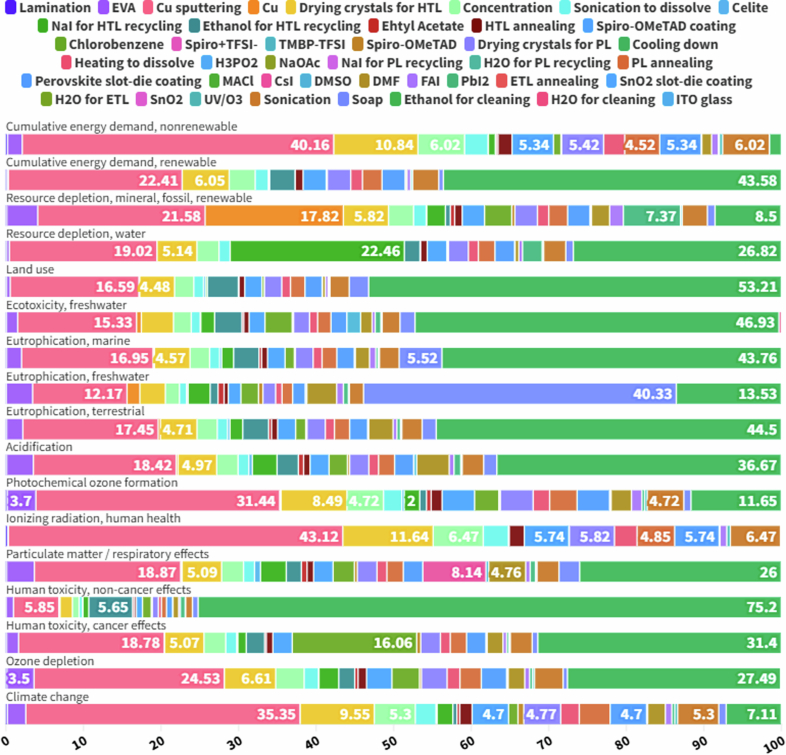
Contribution analysis results for the recycling scenario. The perovskite solar cell with a ‘cradle-to-cradle’ system boundary is considered. The figure is generated using the Flourish online tool.

We assess midpoint impact categories following the Product Environmental Footprint (PEF) methodology, as outlined in the International Energy Agency Photovoltaic Power Systems Programme Task 12 report, to reveal the full-spectrum consequences associated with climate change, human health, resource depletion and so on^32^. We find that the environmental performance of the proposed recycling method outperforms those of the landfilling scenario, for all evaluated indicators (Fig. 4b). Specifically, our recycling strategy can greatly reduce the human toxicity associated with perovskite solar cells by 68.8% (cancer effects) and 57.6% (non-cancer effects) compared with landfilling. We further compare the environmental performance of landfilled and recycled perovskite PVs with that of landfilled and recycled silicon PVs, as illustrated in Supplementary Fig. 10. The results show that perovskite PVs recycled with the proposed holistic recycling strategy exhibits the lowest environmental impacts. We attribute this merit to the high atomic efficiency of end-of-life recycling that minimizes the emissions, especially for critical metals, such as indium, and toxic lead ions. Moreover, our holistic recycling strategy can recover most of the components in perovskite solar cells, substantially reducing the need for excessive virgin material inputs. Thus, it alleviates the resource depletion problem by 96.6% compared with the landfill scenario (Extended Data Figs. 9 and 10).

For the techno-economic assessment (TEA), we use the levelized cost of electricity (LCOE) metric, which is the average net present cost of electricity generation for a power plant over its lifetime, as the comparing figure for a fair comparison in techno-economic details among different PVs. Our multi-round recycling strategy can effectively reduce the LCOE regardless of system lifetime compared with the landfilling scenario, in both residential and utility-scale cases (Fig. 4c). For instance, the landfilled perovskite PV modules in the utility-scale systems with a 15-year lifetime exhibit an LCOE of 4.99 ± 0.32 cents per kWh (nominal LCOE ± deviation owing to uncertainty), lying between the values of market-leading utility-scale solar PVs at 2.4–9.6 cents per kWh in 2023 (ref. ^33^). Yet, our recycling strategy can further cut its LCOE by 18.8% to 4.05 ± 0.13 cents per kWh with three-time recycling. The LCOE gap between recycling and landfill options becomes larger with a shorter system lifetime (reduction of LCOE by 31.3% for 5-year lifetime utility systems). This is because shorter service time requires more virgin material inputs in landfills in the near term, whereas our recycling strategy can reuse the materials at a small recycling cost without sacrificing the overall power conversion efficiency. The LCOE over an extended system lifetime, accounting for more rounds of recycling, is presented in Supplementary Fig. 11.

## Methods

### Materials

The materials used were as follows: lead iodide (PbI_2_) (>98%, TCI Materials), methylammonium iodide (MAI) (Greatcell Solar), formamidinium iodide (FAI) (Greatcell Solar), NaOAc (Sigma-Aldrich), NaI (Sigma-Aldrich), H_3_PO_2_ (Sigma-Aldrich), EA (Sigma-Aldrich), ethanol (EtOH) (96%, Solveco), spiro-OMeTAD (99.5%) (Xi’an p-OLED), methylammonium chloride (MACl) (Xi’an Polymer Light Technology Corp.), caesium iodide (CsI) (99.999% Sigma-Aldrich), anhydrous N,N-dimethylformamide (99.8%, Sigma-Aldrich), dimethyl sulfoxide (DMSO) (99.9%, Sigma-Aldrich) and n-octylammonium iodide (OAI) (Greatcell Solar).

### Preparation of aqueous solution for perovskite recycling

Three additives (NaOAc, NaI and H_3_PO_2_) were dissolved in deionized (DI) water, assisted by sonication with concentrations of 500 mg ml^−1^ for NaOAc, 800 mg ml^−1^ for NaI and 40 µl ml^−1^ for H_3_PO_2_. For example, 2.5 g of NaOAc was dissolved in 5 ml of DI water, followed by dissolving 4 g of NaI and the addition of 200 µl of H_3_PO_2_, to prepare the recycling solution for MAPbI_3_, FAPbI_3_ and mixed-cation perovskites. Note that the concentration for NaOAc, NaI and H_3_PO_2_ can also be varied in the ranges 100–700 mg ml^−1^, 300–900 mg ml^−1^ and 0.1–10.0 v/v%, respectively, to achieve the recycling of perovskite crystals.

### Recycling of the perovskite

Each end-of-life perovskite module was soaked in the 20 ml aqueous solution at 80 °C for 20 min. After the perovskite layer was dissolved, the remaining part (SnO_2_-coated ITO substrate) was rinsed and removed from the solution. The solution was gradually cooled down at a rate of roughly 10 °C per hour to grow the perovskite crystals. The solid crystal and liquid solution were then separated by centrifuging at 5,000 rpm for 3 min. The obtained crystals were then washed with a mixture of ethanol (70%) and EA (30%) for three times and dried in a vacuum oven at 60 °C for 24 h to eliminate the residue. The effective drying process ensures that the recycling solvent of water does not pose a stability issue for the devices.

### Recycling of spiro-OMeTAD

The de-encapsulated 20 modules were soaked in 10 ml of EA for 3 min. Afterwards, each module was rinsed with 5 ml of EA. Then, tetrabutylammonium iodide (8 mg) was introduced into the EA solution (total volume 110 ml). The solution was sonicated at 50 °C for 5 min, during which time it gradually transitioned from brown to light yellow. Following sonication, the solution was passed through a 0.5-cm-thick silica gel pad for filtration. Subsequently, the filtrate was rotary evaporated under reduced pressure until it reached a final volume of 1 ml. To this concentrated solution, 10 ml of 95% ethanol was added, resulting in a large amount of white solid precipitating from the solution. This suspension was stored in a refrigerator at 4 °C for 20 min and then centrifuged at 4,000 rpm for 3 min. The supernatant was carefully decanted and the white solid at the bottom was washed once with ethanol. Next, the white solid was dried in a vacuum oven at 50 °C for 2 h.

### Recycling of the electrode

The degraded solar cells were collected and placed on the hotplate (preheated to 150 °C) for 3 min to soften EVA encapsulant and the cover glasses were then delaminated. After spiro-OMeTAD was dissolved with EA, the brown solution (about 110 ml) was centrifuged at 4,000 rpm for 5 min to collect the bottom-electrode powder. The electrode powder was washed twice with 10 ml of ethanol by sonicating for 10 min, centrifuging at 4,000 rpm for 5 min for each washing process and finally dried in a vacuum oven at 60 °C for 2 h. The collected electrode powder was directly added to the evaporation boat for recycled device fabrication without further treatment. The recycling ratio of the electrode was determined from the weight of the recycled electrode powder and the calculated amount of the electrode on the device from thickness (80 nm), device area and electrode density. The measured recycling ratio was 96.8%, which provided experimental data for life-cycle inventory (LCI) development.

### Recycling of the SnO_2_ + ITO substrate

After perovskite recycling, the ITO + SnO_2_ substrates were sonicated in the 10 ml water/ethanol (50%/50% volume ratio) mixture for 15 min to remove the residue. Then the substrates were dried with a compressed nitrogen flow and treated with ultraviolet–ozone for 15 min.

### Holistic recycling and multi-round recycling

The holistic recycling was conducted by a layer-by-layer recycling for the cover glass, electrode, spiro-OMeTAD, perovskite and SnO_2_ + ITO substrate as described above. The multi-round recycling was conducted by a repeated degradation–recycling process. The unencapsulated fresh devices were exposed to an accelerated degradation process at 85 °C with a relative humidity of 60% under AM 1.5G conditions until the device efficiency loss exceeded 20%. The end-of-life devices (about 400 substrates, including approximately 2,400 working pixels fabricated with the same composition) were holistically recycled as described above to fabricate the recycled devices. During the refabrication of recycled devices, the residue solution (for both spiro-OMeTAD and perovskite layer) on the wall of the spin coater was collected and reused to improve the utilization ratio. The recycled devices were degraded under the accelerated condition as before (85 °C with a relative humidity of 60% under AM 1.5G conditions) and then recycled. This procedure was repeated until the fifth-round recycled devices were completed. As the utilization ratio of the evaporation process is relatively low (<10%), extra electrode materials (100 mg in total for five rounds of recycling) were added to the recycled electrode to ensure a sufficient amount of materials for device refabrication. It further highlighted the importance of improving utilization ratios in device fabrication.

### Precursor preparation

The control perovskite FA_0.97_Cs_0.03_PbI_3_ active layer precursor was prepared by dissolving 1.85 M lead iodide, 1.65 M FAI, 0.58 M MACl and 0.05 M CsI in 1 ml DMF and DMSO mixed solution at a volume ratio of 8:1. The recycled perovskite precursor was prepared by dissolving 1.65 M recycled FAPbI_3_ powder, 0.2 M PbI_2_, 0.58 M MACl and 0.05 M CsI in 1 ml DMF and DMSO mixed solvent (volume ratio 8:1).

The ion-modulated radical doping of spiro-OMeTAD was prepared as previously reported by mixing 90 mg ml^−^^1^ spiro-OMeTAD in chlorobenzene solution with 5 mol% spiro-OMeTAD^2·+^(TFSI^−^)^2^ and 8 mol% EDMPA^+^TFSI^−^ (ref. ^34^).

### Device fabrication

All ITO substrates were cleaned sequentially in DI water and ethanol for 15 min, respectively, and dried by a compressed nitrogen gun. After 15 min of ultraviolet–ozone surface treatment, the SnO_2_ electron transport layer was deposited by spin-coating a 1:6 diluted SnO_2_ nanoparticle aqueous solution (Alfa Aesar) at 4,000 rpm for 30 s, followed by annealing at 150 °C for 20 min in air. The perovskite layer was then deposited by means of spin-coating the perovskite precursor at 5,000 rpm for 30 s. Within 10 s of the 5,000-rpm spinning, 100 µl of chlorobenzene as the antisolvent was dropped onto the film. After spin-coating, the perovskite film was then annealed at 150 °C for 15 min in ambient air. Then 5 mg ml^−1^ OAI solution in IPA was spin-coated onto the perovskite surface at 5,000 rpm and annealed at 100 °C for 3 min for the surface passivation. Subsequently, the hole transport layers were deposited by spin-coating at 5,000 rpm for 30 s without further annealing. A metal electrode (80-nm Au) was finally deposited through the thermal evaporation method under a vacuum degree higher than 3 × 10^−^^6^ Torr to accomplish the solar cell fabrication. We used a 0.06-cm^2^ shadow mask to define the effective working area of the solar cells.

### Device stability measurements

The recycled and fresh devices for stability measurements were fabricated following the same recipe. They were placed in an ageing box for one-sun light soaking in N_2_ or thermal stressed in an oven set to 85 °C in ambient air following the suggested procedure^35^.

### DFT calculations

We carried out DFT calculations using the VASP code with projector augmented-wave potentials^36–38^. We used a plane-wave energy cutoff of 500 eV and a single Γ k-point for the molecular calculations. The exchange-correlation interactions were treated with the generalized gradient approximation of the Perdew–Burke–Ernzerhof (PBE) parametrization^39^. Grimme’s D3 correction was also included to deal with the van der Waals interactions^40^. We used a cubic box with length 20 Å for molecular calculations and a 8 × 8 × 5 k-mesh for the calculation of the PbI_2_ unit cell. The reaction equation for sodium iodine addition is Pb_2_I_4_L_6_ + I^−^ → [Pb_2_I_5_L_6_]^−^.

### LCA modelling

In our LCA work, we have methodically assessed various environmental impacts such as cumulative energy demand, carbon emissions and a comprehensive range of effects using the PEF approach^32,41–49^. The system boundaries were carefully defined to encompass the stages of raw material acquisition, module production and their subsequent recycling. The functional unit for our analysis is defined as one square metre of module area, aligning with industry standards for solar panel life-cycle analysis, despite not mirroring the dimensions of commercially available solar panels^50,51^.

We have compiled an exhaustive LCI dataset, which covers detailed material and energy flows at every stage of the life cycle for the perovskite solar cell under study (Supplementary Tables 1–12). In this work, copper is considered as a replacement for noble metals (gold or silver) to ensure that our assessment reflects future industry-relevant production processes, as has been done in the previous LCA report^41^. The calculations for material and energy use are based on experimental data from the fabrication and recycling processes (including both the utilization ratio and the recycling ratio of materials according to the literature^42^) of these perovskite solar cells. All equipment involved in the fabrication and recycling of perovskite solar cells operates on electricity, which is factored into the life cycle inventory^43,52^. The electrical consumption for each process is estimated by multiplying the power use by the operation duration throughout the solar cell fabrication and recycling stages. Although this approach may lead to an overestimation of the energy required for fabricating and recycling solar cells, it remains appropriate for this assessment. Given the substantial life-cycle environmental impacts embedded in materials compared with energy use, this method effectively demonstrates the environmental benefits of material retention through recycling as outlined in this study. Furthermore, for processes projected to scale up to industrial production, our estimations for energy consumption and material use are extrapolated from the existing literature, ensuring relevance and applicability^53^.

During the evaluation phase of our life-cycle impact assessment (LCIA), we have categorized the results according to various impact metrics, as per the chosen LCIA methodology. We used the PEF to reveal a comprehensive environmental profile of the perovskite solar cell being analysed. The LCIA data for the perovskite solar cell provided valuable insights into how the materials used and the processes involved contribute to different environmental impact indicators. The variations in sustainability metrics, according to the recycling frequencies, were analysed to align with specific environmental load lifetimes. These analyses emphasize the importance of considering both lifespan and recycling times when assessing the economic efficiency of perovskite solar cells.

### Levelized cost of electricity

The LCOE is defined as the ratio of the total lifetime cost to the lifetime electricity production as follows^42,54,55^: ${\rm{L}}{\rm{C}}{\rm{O}}{\rm{E}}=\frac{{\rm{C}}{\rm{I}}+{\sum }_{t=0}^{N}\frac{{\rm{O}}{\rm{M}}(t,d)}{{(1+r)}^{t}}}{{\sum }_{t=0}^{N}\frac{E(t,d)}{{(1+r)}^{t}}}$. In the economic analysis of perovskite solar cells, CI denotes the upfront capital required to deploy the system, encompassing expenses related to the perovskite modules, installation labour, balance of system, inverters and permits, among other factors. OM denotes the annual operation and maintenance and module rejuvenation costs in year *t*. *E* stands for the annual electric power generated by the system in year *t*. *N* describes the expected service life of the PV system, whereas *d* indicates the rate at which the modules degrade each year. The discount rate, which is used to adjust future costs to the present value, is represented by *r*, following the precedent set by the existing literature^55–57^. These calculations take into account costs that are directly tied to the size of the system, the power output and a set initial investment for each project.

### Recycling cost estimation

To estimate the cost of the recycled module, we aggregate various expenses, including utilities, labour, depreciation, maintenance and materials. Details about equipment costs, electricity consumption, process throughput and labour expenses are derived from the existing literature, facilitating our techno-economic analysis^58–60^ (Supplementary Table 10). Maintenance costs for the facilities are projected to be around 20% of the annual depreciation of the equipment. Finally, material costs are estimated by combining insights from the existing literature with quotes from material providers.

## Online content

Any methods, additional references, Nature Portfolio reporting summaries, source data, extended data, supplementary information, acknowledgements, peer review information; details of author contributions and competing interests; and statements of data and code availability are available at 10.1038/s41586-024-08408-7.

## Supplementary information

Supplementary Information This file contains 11 Supplementary Figures (recycling process, materials and devices characterizations, environmental impacts compared with silicon and LCOE with varied lifetime and recycling cycles), four Supplementary Notes (discussion of the recent progress in perovskite recycling, DFT calculations, repairing function of the aqueous recycling solution and green-solvent-based spiro-OMeTAD recycling), 12 Supplementary Tables (LCI) and 27 Supplementary References.

Supplementary Video 1 Aqueous-based recycling of perovskite. This video shows the simple process of recycling perovskite with the aqueous solution.

## Source data

Source Data Fig. 1

Source Data Fig. 2

Source Data Fig. 3

Source Data Fig. 4

Source Data Extended Data Fig. 3

Source Data Extended Data Fig. 5

Source Data Extended Data Fig. 6

Source Data Extended Data Fig. 7

## Data Availability

All data needed to evaluate the conclusions in this paper are present in the paper and/or the Supplementary Materials. Further data are available from the authors on request. Source data are provided with this paper.
