# Supplementary material for: Aqueous-based recycling of perovskite photovoltaics
Source: Nature. 2025 Feb 12;638(8051):670–5. doi: 10.1038/s41586-024-08408-7 (PMC11839462; doi:10.1038/s41586-024-08408-7)
Supplement: Supplementary file 1 — This file contains 11 Supplementary Figures (recycling process, materials and devices characterizations, environmental impacts compared with silicon and LCOE with varied lifetime and recycling cycles), four Supplementary Notes (discussion of the recent progress in perovskite recycling, DFT calculations, repairing function of the aqueous recycling solution and green-solvent-based spiro-OMeTAD recycling), 12 Supplementary Tables (LCI) and 27 Supplementary References. [file 41586_2024_8408_MOESM1_ESM.pdf]

nature portfolio

https://doi.org/10.1038/s41586-024-08408-7

Supplementary information

Aqueous-based recycling of perovskite photovoltaics

In the format provided by the authors and unedited

Supplementary Information for

Aqueous based recycling of perovskite photovoltaics

Xun Xiao[1], Niansheng Xu[1], Xueyu Tian[2], Tiankai Zhang[1], Bingzheng Wang[2], Xiaoming Wang[3,4], Yeming Xian[3,4], Chunyuan Lu[5,6,7], Xiangyu Ou[1], Yanfa Yan[3,4], Licheng Sun[5,6,7,8], Fengqi You[2,9,10*] and Feng Gao[1,5,6,11*]

[1]*Department of Physics, Chemistry and Biology, Linköping University, Linköping, Sweden*

[2]*Systems Engineering, College of Engineering, Cornell University, Ithaca, NY, USA*

[3]*Department of Physics and Astronomy, The University of Toledo, Toledo, Ohio 43606, USA*

[4]*Wright Center for Photovoltaics Innovation and Commercialization, The University of Toledo, Toledo, Ohio 43606, USA*

[5]*Center of Artificial Photosynthesis for Solar Fuels, Westlake University, 310024, Hangzhou, China*

[6] *Department of Chemistry, School of Science, Westlake University, 310024, Hangzhou, China*

[7]*Research Center for Industries of the Future, Westlake University, 310024, Hangzhou, China*

[8]*Division of Solar Energy Conversion and Catalysis at Westlake University, Zhejiang Baima Lake Laboratory Co., Ltd, Hangzhou 310000, Zhejiang Province, China*

[9]*Robert Frederick Smith School of Chemical and Biomolecular Engineering, Cornell University, Ithaca, NY, USA*

[10]*Cornell Atkinson Center for Sustainability, Cornell University, Ithaca, NY, USA*

[11]*Wallenberg Initiative Materials Science for Sustainability, Department of Physics, Chemistry and Biology, Linköping University, Linköping, Sweden*

[*] Correspondence to F.G. (email: feng.gao@liu.se) and F.Y. (email: fengqi.you@cornell.edu).

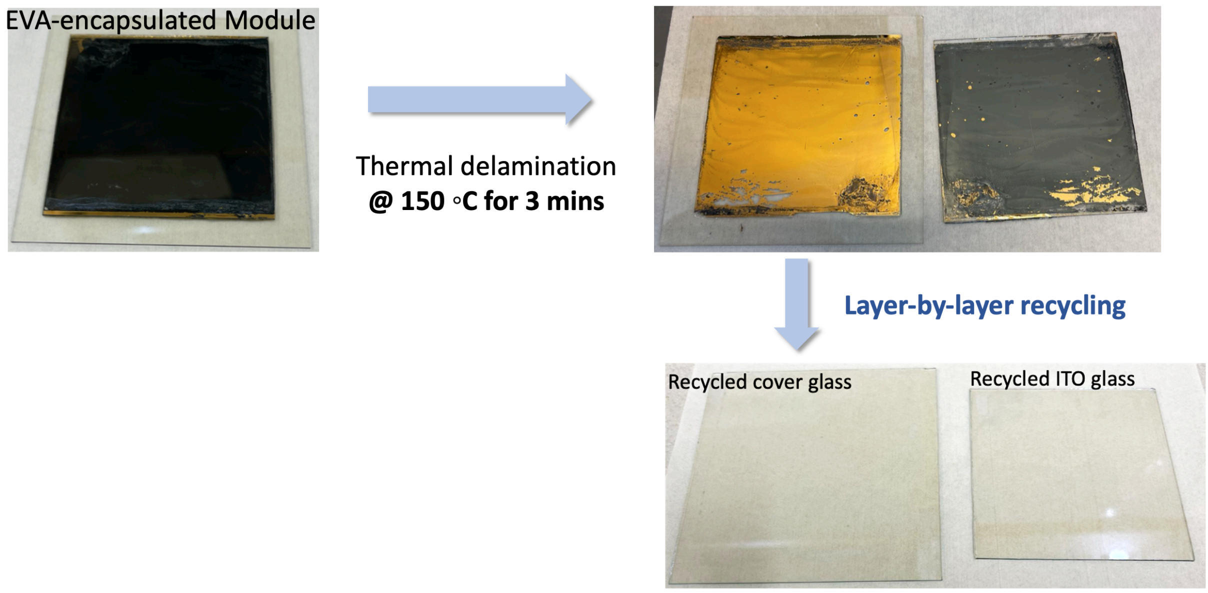

Supplementary Figure 1. Recycling process of a degraded module with EVA encapsulation. Thermal delamination was applied by 150 °C for 3 mins to separate cover glass and device area. Then a layer-by-layer recycling procedure was conducted to achieve a holistic recycling.

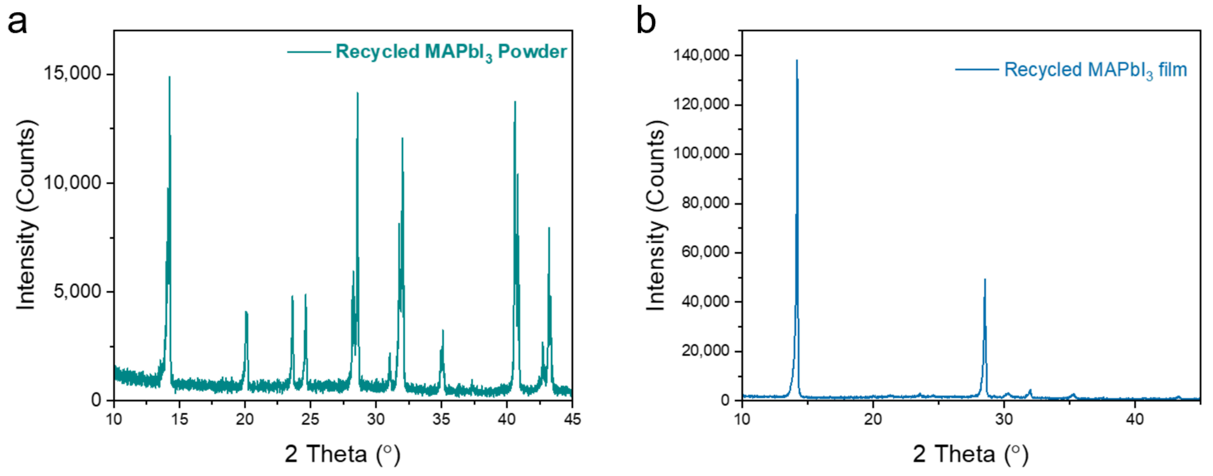

Supplementary Figure 2. XRD pattern of recycled $MAPbI_3$ powder (**a**) and film made with recycled perovskite crystals (**b**).

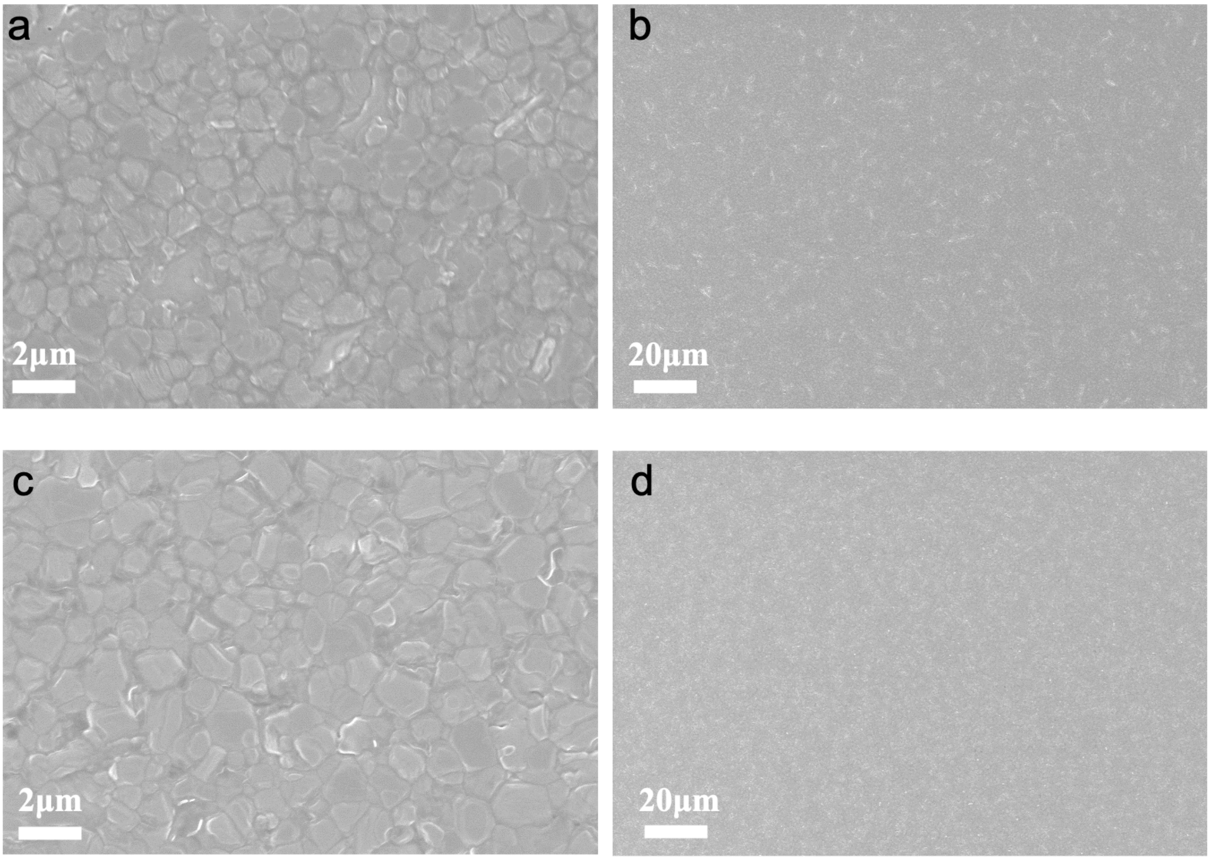

Supplementary Figure 3. Top surface SEM images of fresh $FAPbI_3$ thin films (**a** & **b**) and recycled films (**c** & **d**).

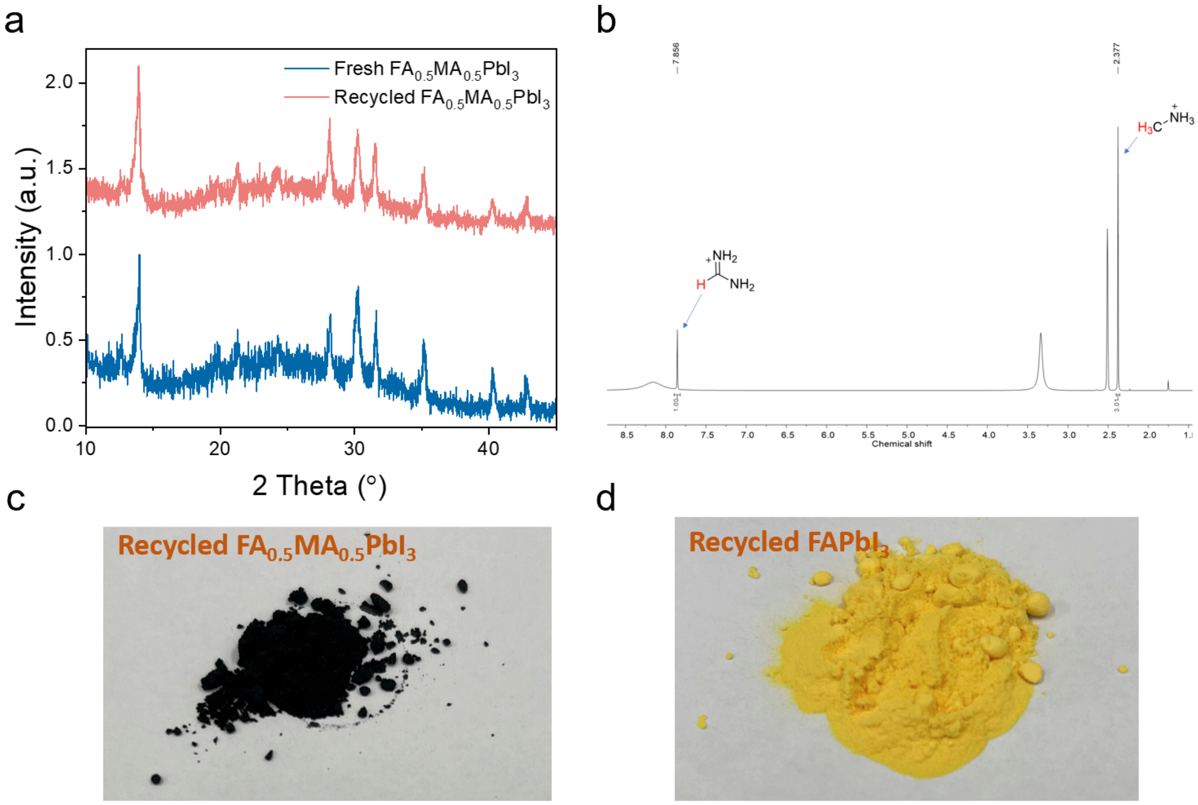

Supplementary Figure 4. a. XRD pattern of recycled $FA_{0.5}MA_{0.5}PbI_3$ film made with fresh raw materials and recycled perovskite crystal powders; **b.** ^{1}H-NMR of recycled $FA_{0.5}MA_{0.5}PbI_3$ powder, where the ratio of MA:FA can be confirmed to be 1:1; **c.** image of recycled $FA_{0.5}MA_{0.5}PbI_3$ crystal powder; **d.** image of recycled $FAPbI_3$ crystal powder.

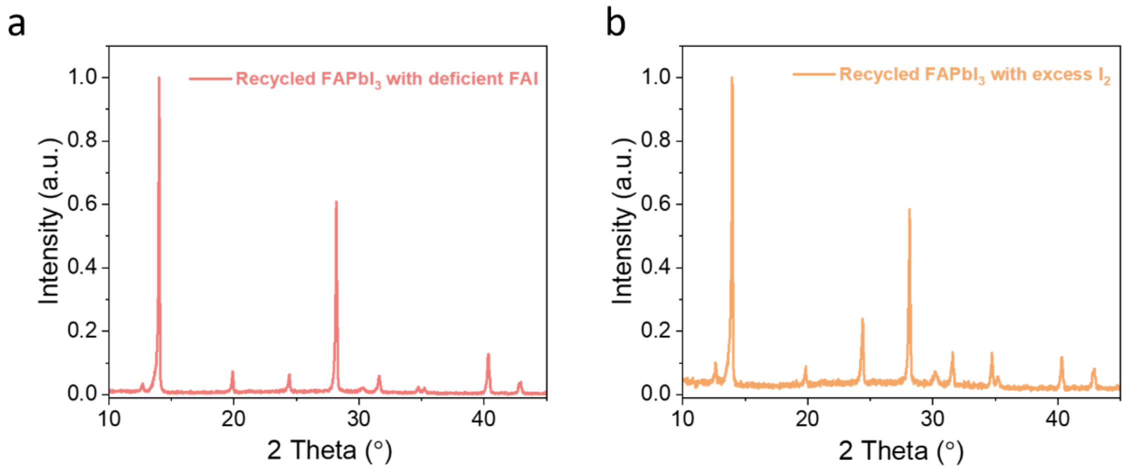

Supplementary Figure 5. XRD pattern of recycled $FAPbI_3$ film from $FAPbI_3$ raw materials with 10% FAI deficiency (**a**) and 10% I_2 excess (**b**), respectively.

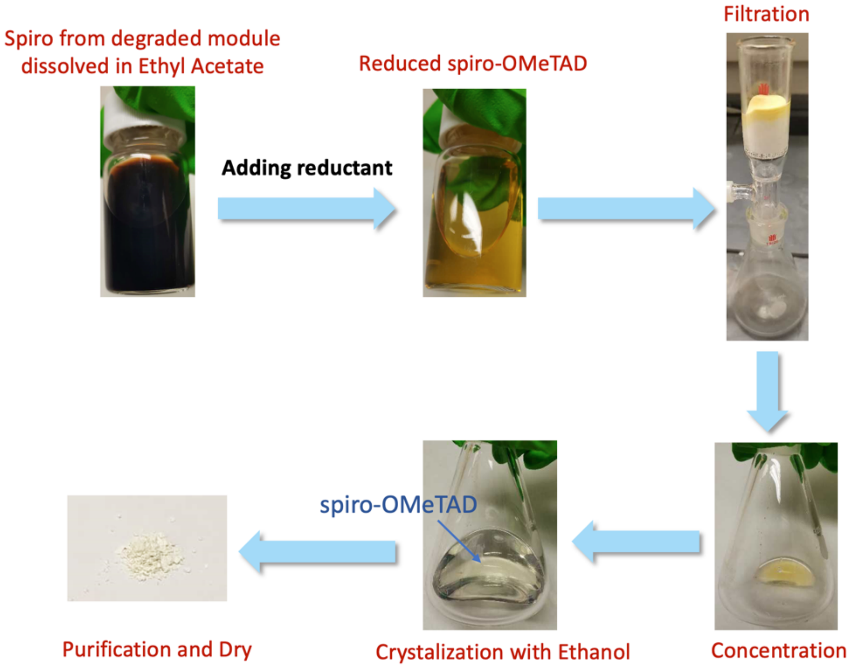

Supplementary Figure 6. Recycling process of Spiro-OMeTAD.

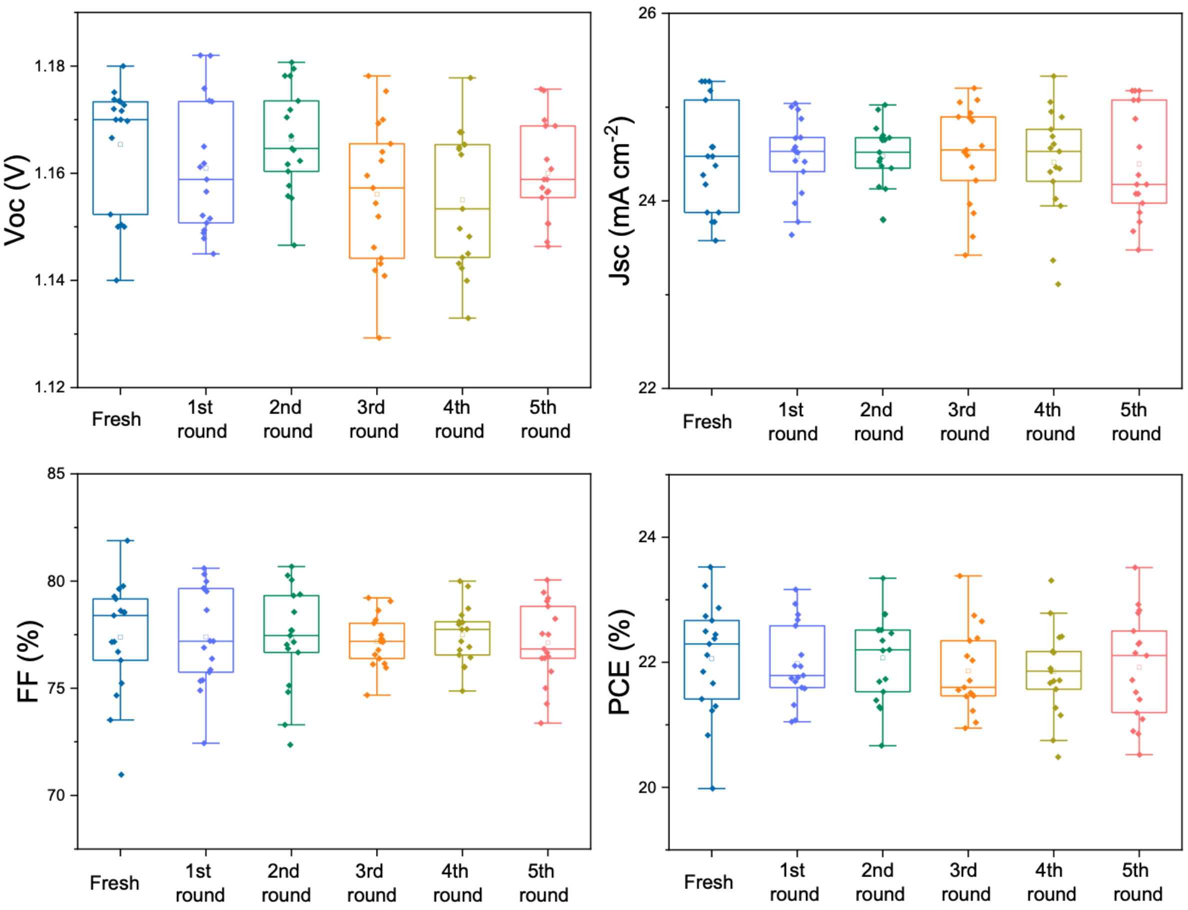

Supplementary Figure 7. Statistic results of device performance under multi-round recycling.

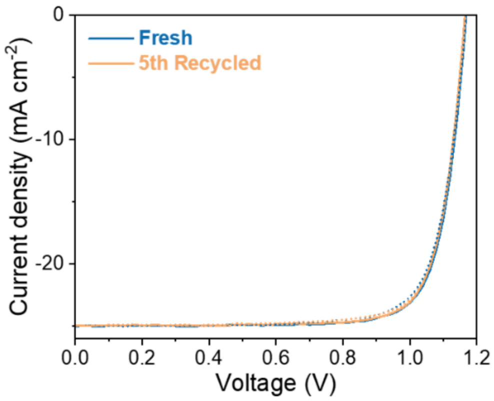

Supplementary Figure 8. J-V curves of fresh and 5th recycled perovskite solar cells. Solid and dot lines are reverse and forward scans, respectively.

a

b

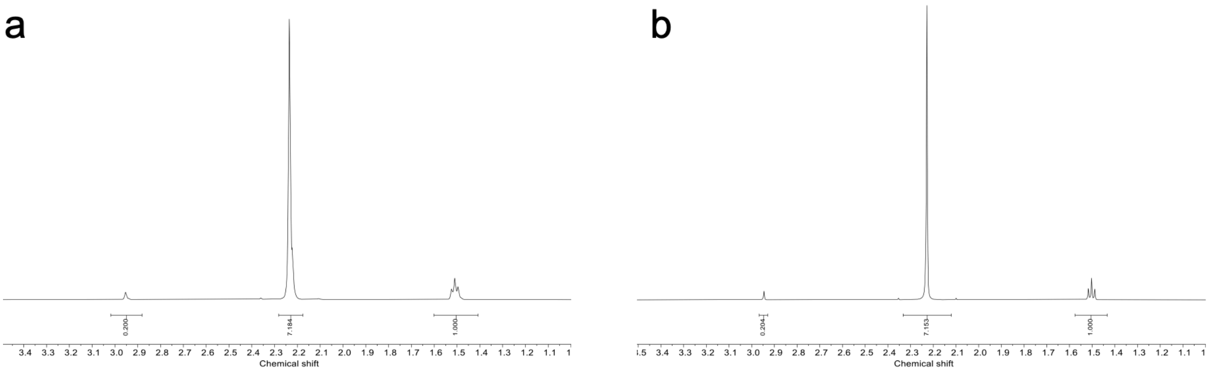

c

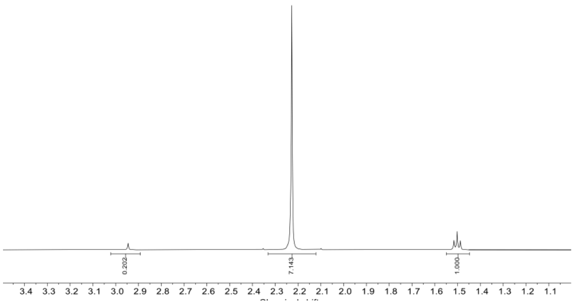

d

	**MA^+ Intensity**	**$Acetate^-$ Intensity**
**1st recycling**	0.200	7.184
**3rd recycling**	0.204	7.153
**5th recycling**	0.202	7.143

Supplementary Figure 9. **a-c** are ^{1}H-NMR of the water solution after 1, 3, and 5 cycles of recycling, where a fixed amount (10 µL per 1 mL) of ethanol is added for calibration; **d** is the summarized integration intensity for the organic ions concentration for different recycling cycles.

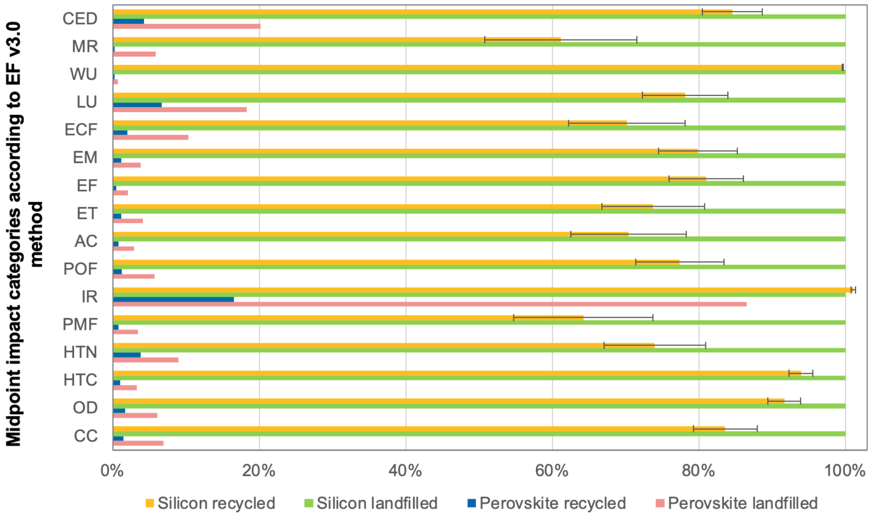

Supplementary Figure 10. Comparison of full-spectrum midpoint impact categories among landfilled perovskite PV, recycled perovskite PV, as well as landfilled and recycled benchmark silicon PV according to EF v3.0 method. The LCI of the silicon recycling process is based on the IEA PVPS Task 12 report.[1] To convert the mass-based functional unit from the reference report to an area-based functional unit for our study, we used the common unit weight range of silicon PV modules, which are approximately 11-19 kg m^{-2}.[2] Specifically, 19 kg m^{-2} corresponds to the lowest functional unit-based environmental impacts, thus representing the lower bounds of the error bar, while 11 kg m^{-2} corresponds to the upper bounds of the error bar. This weight range allows us to account for the variability in the impact of silicon PV recycling. While silicon recycling can significantly reduce the environmental impact of silicon PV fabrication, perovskite solar cell recycling still demonstrates a substantially lower environmental impact compared to silicon PV recycling.

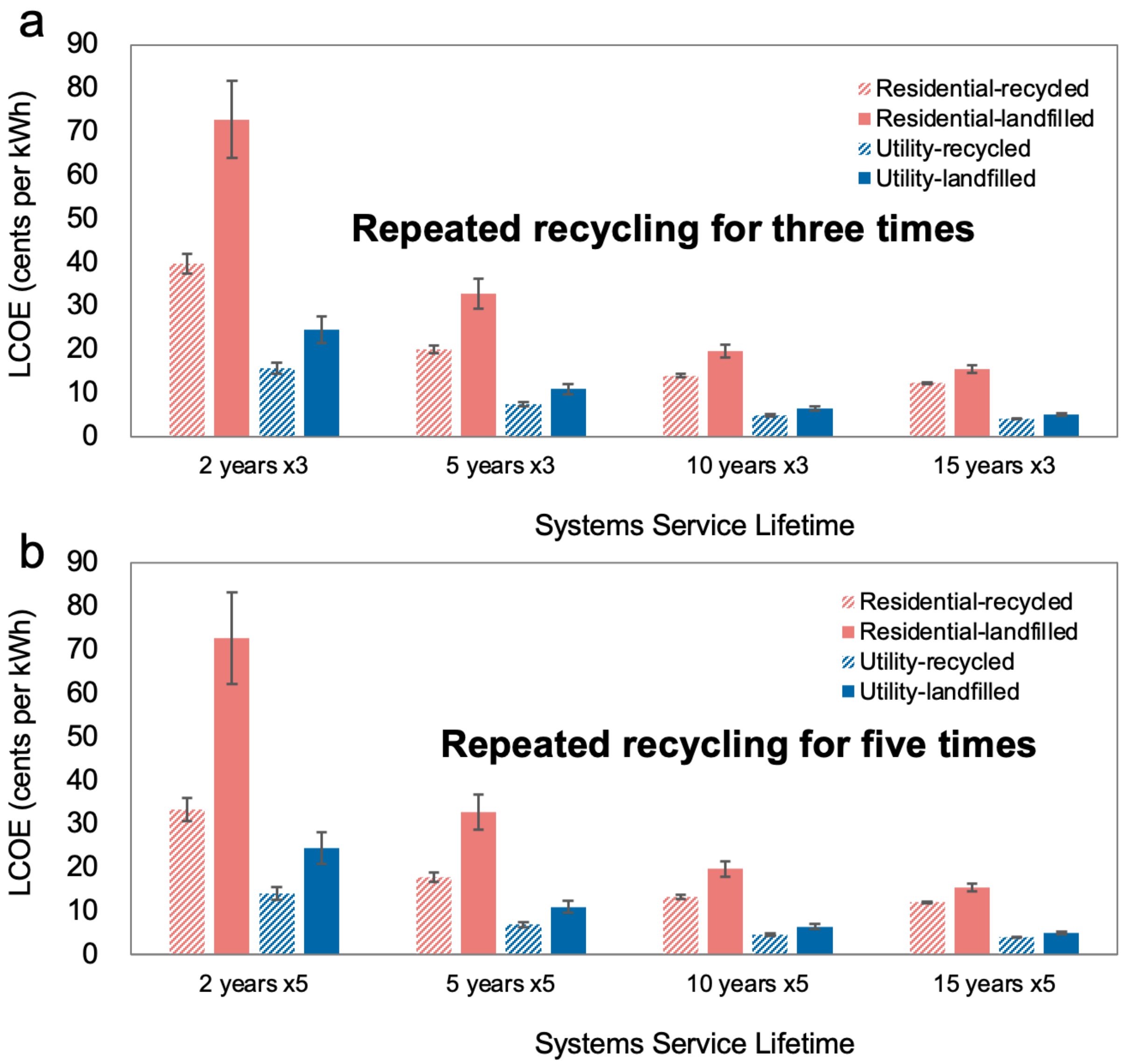

Supplementary Figure 11. LCOE of perovskite PV with varied device lifetime and recycling cycles. The error bars illustrated in the graph convey the uncertainty in the LCOE estimates, reflecting a ±20% fluctuation in crucial input factors. This variability includes the expenses linked to energy consumption, labor, materials, and equipment, all integral to the processes of recycling, remanufacturing, and re-installing PV modules.

Supplementary Note 1: Recent progress in perovskite solar module recycling

Lead recycling from degraded perovskite modules has been recently investigated to address the toxicity concern.[3,4] Typically, lead ions are extracted from the dissolved degraded perovskite with lead absorbents in dimethylformamide (DMF) solution, and subsequently recovered to high-quality lead iodide by introducing iodine. The lead recycling efficacy is optimized high, yet the atomic recycling efficacy is low.[3,4] For example, the iodine elements, accounting for ~60 wt% in perovskites, are not fully recycled.[3] Note that, reserved iodine on the upper earth crust is even less abundant than the rare earths, and the long-term availability of iodine and cesium is a rising concern for large-scale deployment of perovskite PVs.[5-7] A sequential recycling strategy, which involves layer-by-layer dissolution of the functional layers with processing solvents, i.e. DMF and chlorobenzene (CB), and then redeposition of the layers to build a perovskite solar cell device, have been developed to improve the atomic recycling efficacy.[8-10] However, it lacks chemical treatment to repair degraded materials, leading to low-quality recycling products from end-of-life perovskite solar modules.

Moreover, while the recycling processes are designed for environmental protection, state-of-the-art recycling strategies, relying on hazardous solvents like DMF, CB, or methylamine, raise significant environmental concerns.[11-13] It also brings compatibility issues with industrial processes.[14-16] While green solvents like bio-derived γ-valerolactone, etc, have been explored in device fabrication, the recycling methods with green solvents and high-efficiency are rarely discussed.[17,18]

In parallel with these experimental efforts, a recent holistic LCA study was conducted on the recycling processes of perovskite solar cells based on the "best available" experimental data at that time, primarily focusing on the recovery of electrode and substrate materials with approaches involving hazardous solvents like DMF.[19]

Supplementary Note 2: DFT calculation of ion-assisted phase changes in water solution

With the introduction of sodium acetate, the intercalated acetate ions would attack the PbI_2 layers through Lewis acid-base bonds (-C=O-Pb-) formation[20], breaking the Pb-I bonds and forming the water-soluble octahedral structure **i** (**Fig. 1d**). The reaction can be denoted as $PbI_2 + 4L \rightarrow PbI_2L_4$ with an energy gain of 0.20 eV, where L refers to the acetate ions.[21] The octahedron would be connected by releasing two acetate ions and sharing two iodine ions, resulting in a water-soluble edge-sharing structure **ii**. The reaction equation is $2PbI_2L_4 \rightarrow Pb_2I_4L_6 + 2L$ with an energy gain of 0.72 eV. Thus, the acetate ions could facilitate PbI_2 dissolution in water with a reaction of $2PbI_2 + 6L \rightarrow Pb_2I_4L_6$ for 1.12 eV energy gain. With sodium iodide added into the solution, the edge-sharing octahedral configuration is transformed into corner-sharing structure **iii**, gaining extra energy of 0.23 eV. This corner-sharing octahedral configuration serves as the building block of metal halide perovskites to be precipitated.[21]

Supplementary Note 3: Repairing and recycling degraded perovskite with water solution

As end-of-life solar panels awaiting recycling are degraded, the ability to repair the degraded perovskite is critical in the recycling process. Since FAI deficiency and excess I_2 are two common situations of the $FAPbI_3$ degradation, we mimic the degraded $FAPbI_3$ by a 10% FAI deficiency or an additional 10 wt% I_2 to the stoichiometry $FAPbI_3$ perovskite. Following the aforementioned procedure, we recycle the mimicked degraded perovskites and utilize the recycled crystals to fabricate thin films for measurements. In these films, we obtain almost identical high-crystalline XRD peaks to those observed in fresh films (**Supplementary Fig. 5**), indicating the repairing capacity of our recycling processes. We attribute the repair capacity to the fact that the I_2 can be effectively reduced/repaired to I^- for crystal growth with the aid of H_3PO_2 and that the crystal growth process during recycling can automatically rectify deficiencies and defects formation. It is worth noting that the ion species and concentration of the solution can be readily monitored and restored during high-volume production in the practical fabrication process.

Supplementary Note 4. Green solvent recycling of spiro-OMeTAD

Initially, the spiro-OMeTAD on the end-of-life module is extracted by selectively dissolving it with ethyl acetate (EA). Spiro-OMeTAD in efficient solar cells is doped, and impurities within the EA solution include the spiro-OMeTAD radical-cation and salt-based dopant additives. The solution appears dark brown, owing to the color of the narrow gap spiro-OMeTAD radical-cation, as shown in **Supplementary Fig. 6**. Thus, we implement a reduction process using reductants such as tetrabutylammonium iodide to convert it to a neutral state via the following reaction.

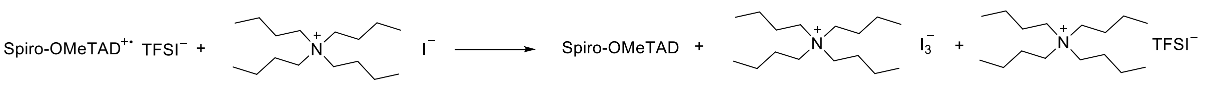

Upon addition of tetrabutylammonium iodide, the solution turns light yellow due to the color of I_3^-. Tetrabutylammonium iodide is chosen as a reducing agent because its by-products, tetrabutylammonium triiodide and tetrabutylammonium TFSI, are highly soluble in hydrophilic solvents such as ethanol, facilitating their easy removal during the precipitation process. Moreover, a simple filtration step with silica gel is employed to eliminate the EVA impurity from the solution, as a small amount of EVA dissolution in EA is observed during extraction of spiro-OMeTAD (**Extended Data Fig. 6a**). The silica gel can strongly interact with the large amount of polar acetate units in EVA to absorb the EVA impurity. Following this, we concentrate the solution, and add hydrophilic anti-solvent, ethanol (EtOH), to induce precipitation of hydrophobic spiro-OMeTAD. Further purification and drying processes using anti-solvent EtOH are applied to achieve the white powder of high-purity neutral state spiro-OMeTAD. Both fresh and final recycled spiro-OMeTAD samples are examined with ^{1}H nuclear magnetic resonance (NMR) spectrum, showing no impurity peaks (**Extended Data Fig. 6b&c**). The radical-cation, i.e. bis(trifluoromethane)sulfonimide (TFSI$^-$), is confirmed to be removed in recycled spiro-OMeTAD with negligible fluoride signal in ^{19}F-NMR spectra (**Extended Data Fig. 6d**).

Life cycle inventories: Supplementary Table 1-9 exhibits the relevant life cycle inventories of perovskite production and recycling. Supplementary Table 1 and 2 show the materials and energy consumption for 1 m^2 perovskite solar cell production. Supplementary Table 3 and 4 describe the energy and materials inventories of 1 m^2 perovskite solar cell recycling process. Supplementary Table 5 shows the key parameters during the recycling process. Supplementary Table 6-9 exhibit the production inventories for per unit FAI, PbI_2, CsI, and spiro-OMeTAD. Supplementary Table 10 shows the breakdowns of direct manufacturing cost for perovskite solar modules. Supplementary Table 11 and 12 show the characterization factors for production extracted from the Eco-invent database[22]

Supplementary Table 1. Material inventory of 1 m^2 of the perovskite solar cell.

Process	Value (kg)	Notes
**Substrate**		
ITO glass	1.54	Substrate
H_2O	0.15	Substrate cleaning solvents
EtOH	0.12	
Soap	0.003	
**ETL Deposition**		
SnO_2	1.86E-4	
H_2O	6.8E-03	Solvent
**Perovskite Layer Deposition**		
PbI_2	4.3E-03	
FAI	1.56E-03	
DMF	5.73E-03	Solvent
DMSO	6.27E-04	Solvent

CsI	6.67E-05	
MACl	3.1E-05	
**HTL Deposition**		150 nm of HTL
Spiro-OMeTAD	2.8E-04	
TMBP-TFSI	11.3E-06	
Spiro+TFSI	2.27E-05	
Chlorobenzene	5.47E-03	Solvent
**Cathode Deposition**		
Cu	12.9E-04	
**Encapsulation**		
EVA	1.00E-02	

Supplementary Table 2. Energy inventory of 1 m^2 of the perovskite solar cell.

Process	Power (W)	Time (s)	Electricity (kWh)	Notes
**Substrate**				
Sonication	1000	1200	0.3333	UIP1000hdT
UV/O_3	138	600	0.0230	
**ETL Deposition**				
SnO_2 slot-die coating	17740	60	0.2957	1 m/min
Annealing	1000	600	0.1667	Electric hot plate 729659 C
**Perovskite Layer Deposition**				
Perovskite slot-die coating	17740	60	0.2957	1 m/min
Annealing	1000	900	0.2500	
**HTL Deposition**				
Spiro-OMeTAD coating	17740	60	0.2957	1 m/min
Annealing	600	600	0.1000	
**Electrode deposition**				
Cu Sputtering	80000	100	2.222	
**Encapsulation**				

Lamination	1500	30	0.0125

Supplementary Table 3. Material inventory for the recycling process of 1 m^2 of the perovskite solar cell.

Process	Value (kg)	Notes
**Substrate**		
H_2O for cleaning	1.50E-01	Substrate cleaning solvents
EtOH for cleaning	1.20E-01	
Soap	3.00E-03	
**Perovskite Layer Deposition**		
PbI_2	1.11E-03	
FAI	4.02E-04	
DMF	5.73E-03	Solvent
DMSO	6.27E-04	Solvent
CsI	1.72E-05	
MACI	7.98E-06	
Deionized water	1.10E-02	100% reusable
NaI	8.60E-03	100% reusable
NaOAc	5.38E-03	100% reusable
H_3PO_2	1.50E-03	100% reusable
**HTL Deposition**		
Spiro-OMeTAD	7.46E-05	
TMBP-TFSI	3.01E-06	
Spiro+TFSI	6.05E-06	
Chlorobenzene	5.47E-03	Solvent
Ethyl Acetate	6.31E-03	80% reusable
Ethanol	9.00E-03	

NaI	5.00E-03
Celite	5.00E-02
**Cathode Deposition**	
Cu	3.53E-04
**Encapsulation**	
EVA	1.00E-02

Supplementary Table 4. Energy inventory for the recycling process of 1 m^2 of the perovskite solar cell.

Process	Power (W)	Time (s)	Electricity (kWh)	Notes
**Substrate**				
Sonication	1000	1200	0.333	UIP1000hdT
UV/O_3	138	600	0.023	
**Perovskite Layer Deposition**				
Perovskite slot-die coating	17740	60	0.296	1 m/min
Annealing	1000	900	0.250	
Heating to dissolve	600	900	0.150	
Drying crystals	600	1800	0.300	
**HTL Deposition**				
Spiro-OMeTAD coating	17740	60	0.296	1 m/min
Annealing	600	600	0.100	
Sonication to dissolve	1000	600	0.167	
Concentration	1000	1200	0.333	
Drying crystal	600	3600	0.600	
**Electrode deposition**				
Cu Sputtering	80000	100	2.222	

**Encapsulation**			
Lamination	1500	30	0.013

Supplementary Table 5. Key parameters of the fabrication and recovery processes.

Parameter	Value
Slot-die material usage efficiency	>90%
Expected Efficiency used for 1 m^2 module	22%
Aperture area geometric factor	93%
ETL utilization rate	75%
Perovskite layer utilization rate	75%
Perovskite layer recycling efficiency	99.0±0.4 wt%
HTL utilization rate	75%
HTL recycling efficiency	97.8±0.3 wt%
Cathode utilization rate	75%
Cathode recycling efficiency	96.8%
Fresh devices efficiency (PCE)	22.1±0.9%
5th recycled devices efficiency (PCE)	21.9±1.1%

Supplementary Table 6. Life cycle inventory for the production process of 1 kg FAI[23].

	Value	Unit
**Process input**		
H_2O	5.763E+01	kg
iodine	3.442E+01	kg
H_2S	4.611E+00	kg
chloroform	1.828E+02	kg
Na	7.721E+01	kg
HOAc	2.039E+01	kg
NH_3	7.560E+00	kg
Ethanol	9.581E+02	kg
cooling	5.729E+00	MJ
heat	9.754E+02	MJ
electricity	1.053E+05	kWh
**Process output**		
$NH{=}CHNH_3I$	1.000E+00	kg
H_2	3.357E+00	kg
NaCl	1.963E+02	kg
Ethanol (chloroform)	7.460E+02	kg
wastewater	1.129E+02	kg
waste	2.114E+02	kg
Ethanol (emitted into the air)	7.171E+01	kg

Supplementary Table 7. Life cycle inventory for the production process of 1 kg PbI_2[19,24].

	Value	Unit
**Process input**		
iodine	6.700E-01	kg
KOH	2.910E-01	kg
Pb	4.490E-01	kg
HNO_3	7.290E-01	kg
heat	1.350E+01	MJ
power	1.330E-01	kWh
**Process output**		
PbI_2	1.000E+00	kg
KNO_3	4.380E-01	kg
KIO_3	1.350E-01	kg
NO	4.340E-02	kg
waste	1.600E-01	kg

Supplementary Table 8. Life cycle inventory for the production process of 1 kg CsI[19].

	Value	Unit
**Process input**		
Sulphuric acid	7.550E-01	kg
Lime	4.317E-01	kg
Iodine	4.884E-01	kg
Hydrogen	3.849E-03	kg
Deionized water	1.027E+01	kg
Pollucite ore	1.695E+00	kg
Crushing	1.695E+00	kg
Heat	1.289E+01	MJ
Electricity	2.700E-02	kWh
**Process output**		
CsI	1.000E+00	kg
H_2O	9.925E+00	kg
Al_2O_3	1.962E-01	kg
SiO_2	9.250E-01	kg
NaOH	3.040E-01	kg
Waste gypsum	1.332E+03	kg

Supplementary Table 9. Life cycle inventory for the production process of 1 kg spiro-OMeTAD[19].

	Value	Unit
**Process input**		
C_6H_6O	0.724	kg
NaOH	1.63	kg
$C_2H_6O_4S$	0.485	kg
KI	0.837	kg
KIO_3	0.412	kg
CH_4O	2.47	kg
HCl	5.04	kg
H_2	0.0295	kg
CH_3ONa	0.279	kg
C_6H_5Cl	0.966	kg
HNO_3	1.08	kg
H_2SO_4	1.62	kg
Br_2	0.604	kg
Coal tar	0.295	kg
C_2H_6O	1.06	kg
$C_6H_5NH_2$	0.944	kg
$NaNO_2$	0.221	kg
SO_2	0.407	kg
Mg	0.0267	kg
Heat	272	MJ
Electricity	24.3	kWh

Process output

Spiro-OMeTAD	1	Kg
Hydrogen bromide	0.567	Kg
2-Chloronitrobenzene	0.428	Kg
Waste	81.9	Kg

Supplementary Table 10. Breakdowns of direct manufacturing cost for perovskite solar module[25-27].

Component	Depreciation ($/m^2)	Maintenance ($/m^2)	Utility ($/m^2)	Labor ($/m^2)	Material ($/m^2)	Total ($/m^2)	Reference
Back glass	0.11	0.01	0.28	0.32	3.04	3.75	2
Sputter ITO	0.60	0.15	0.43	0.16	1.30	2.64	2
SnO_2	0.05	0.01	0.03	0.03	0.09	0.21	3
Perovskite layer	0.16	0.02	0.04	0.68	0.22	1.13	1
Spiro-OMeTAD	0.14	0.03	0.03	0.22	7.04	7.45	3
Cu	0.11	0.02	0.43	0.16	0.01	0.73	3
Interconnection	0.13	0.02	0.01	0.03	0.01	0.19	1
Edge seal	0.12	0.01	0.00	0.03	3.89	4.05	1
Lamination	0.13	0.01	0.00	0.11	1.54	1.78	1
Front glass	0.11	0.01	0.28	0.32	7.00	7.71	1
Junction box	0.11	0.01	0.00	0.03	7.50	7.64	1

Supplementary Table 11. Characterization factors for production extracted from the Eco-invent database V3.10 (Part I)

	Method	IPCC 2021	EF v3.0	EF v3.0	EF v3.0	EF v3.0	EF v3.0	EF v3.0	EF v3.0	EF v3.0	EF v3.0
	**Category**	**Climate change**	**Ozone depletion**	**Human toxicity: carcinogenic**	**Human toxicity: non-carcinogenic**	**Particulate matter formation**	**Ionizing radiation: human health**	**Photochemical oxidant formation: human health**	**Acidification**	**Eutrophication: terrestrial**	**Eutrophication: freshwater**
	**Indicator**	**Global warming potential (GWP100)**	**Ozone depletion potential (ODP)**	**Comparative toxic unit for human (CTUh)**	**Comparative toxic unit for human (CTUh)**	**Impact on human health**	**Human exposure efficiency relative to u235**	**Tropospheric ozone concentration increase**	**Accumulated exceedance (AE)**	**Accumulated exceedance (AE)**	**Fraction of nutrients reaching freshwater end compartment (P)**
**Required Materials**	**Corresponding materials**	**kg CO_2-Eq**	**kg CFC-11-Eq**	**CTUh**	**CTUh**	**disease incidence**	**kBq U235-Eq**	**kg NMVOC-Eq**	**mol H+-Eq**	**mol N-Eq**	**kg P-Eq**
	**1m² Perovskite Module**										
ITO Glass	indium	1.12E+02	1.49E-06	7.87E-07	1.38E-05	5.64E-06	2.62E+01	8.25E-01	1.06E+00	2.75E+00	1.39E-01
ITO Glass	tin	1.05E+01	9.24E-08	2.70E-08	4.37E-07	1.48E-06	9.32E-01	9.23E-02	1.28E-01	4.01E-01	2.45E-02
ITO Glass	glass cullet, sorted	2.25E-02	1.59E-10	9.11E-11	1.88E-09	1.55E-08	4.47E-04	1.02E-04	7.29E-05	2.79E-04	2.31E-06
ITO Glass	titanium dioxide	6.87E+00	9.57E-08	1.93E-08	3.88E-07	5.83E-07	2.53E-01	2.99E-02	5.85E-02	7.63E-02	2.51E-03
ITO Glass	argon, liquid	2.53E+00	1.71E-08	2.87E-09	1.71E-08	1.09E-07	3.63E-01	7.29E-03	1.22E-02	2.46E-02	1.10E-03
ITO Glass	oxygen, liquid	1.13E+00	6.32E-09	1.18E-09	7.34E-09	5.23E-08	1.08E-01	3.32E-03	5.48E-03	1.14E-02	4.47E-04
SnO_2	tin dioxide	8.96E+00	8.07E-08	2.39E-08	3.68E-07	1.24E-06	7.82E-01	7.76E-02	1.08E-01	3.35E-01	2.04E-02
H_2O	water, deionized	4.98E-04	1.61E-10	2.86E-12	2.60E-11	3.53E-11	3.41E-05	1.75E-06	4.44E-06	5.15E-06	2.01E-07
PbI_2	lead	1.42E+00	3.17E-08	1.29E-08	7.13E-07	1.65E-07	1.10E-01	7.88E-03	3.31E-02	2.29E-02	1.44E-03
PbI_2	iodine	5.62E+00	1.37E-07	1.35E-08	6.47E-08	2.28E-07	1.52E-01	1.44E-02	1.99E-02	3.37E-02	8.94E-04
PbI_2	potassium hydroxide	4.74E+00	5.60E-08	2.96E-08	8.91E-08	2.75E-07	4.44E-01	1.61E-02	2.55E-02	5.22E-02	1.88E-03
PbI_2	nitric acid	2.01E+00	1.08E-08	2.32E-09	9.32E-09	6.64E-08	1.26E-02	2.63E-03	1.05E-02	3.89E-02	2.57E-04
FAI	water, deionized	4.98E-04	1.61E-10	2.86E-12	2.60E-11	3.53E-11	3.41E-05	1.75E-06	4.44E-06	5.15E-06	2.01E-07
FAI	iodine	5.62E+00	1.37E-07	1.35E-08	6.47E-08	2.28E-07	1.52E-01	1.44E-02	1.99E-02	3.37E-02	8.94E-04
FAI	hydrogen sulfide	1.46E+00	1.72E-08	1.84E-08	1.10E-08	1.92E-07	3.80E-02	5.30E-03	2.88E-02	8.61E-03	2.72E-04
FAI	trichloroborane	3.97E+00	1.12E-07	1.85E-08	3.33E-07	2.08E-07	3.37E-01	1.45E-02	2.64E-02	4.65E-02	1.47E-03
FAI	sodium	7.27E+00	6.30E-08	1.28E-08	8.31E-08	3.25E-07	1.15E+00	2.18E-02	3.62E-02	7.15E-02	3.22E-03
FAI	acetic acid	3.30E+00	6.13E-08	6.92E-09	9.30E-08	1.59E-07	2.26E-01	1.24E-02	1.30E-02	2.57E-02	9.27E-04

FAI	ammonia, anhydrous	3.23E+00	3.59E-08	4.69E-09	1.50E-08	1.00E-07	1.95E-02	6.87E-03	8.83E-03	1.74E-02	7.38E-04
FAI	ethanol	2.33E+00	4.91E-08	6.92E-09	2.14E-08	8.94E-08	4.94E-02	1.19E-02	1.13E-02	2.23E-02	1.14E-03
DMF	N, N-dimethylformamide	5.72E+00	9.18E-08	2.19E-08	5.67E-08	3.05E-07	2.88E-01	1.79E-02	2.56E-02	6.61E-02	1.47E-03
DMSO	dimethyl sulfoxide	3.01E+00	6.86E-08	1.40E-08	3.34E-08	2.09E-07	1.36E-01	9.85E-03	2.04E-02	1.90E-02	7.00E-04
CsI	pollucite ore	4.32E-02	2.03E-09	4.37E-10	3.79E-09	1.02E-04	3.03E-03	2.66E-04	3.88E-04	1.18E-03	1.59E-05
CsI	sulfuric acid	1.81E-01	2.33E-09	3.37E-09	4.71E-08	6.04E-08	1.35E-02	1.94E-03	1.14E-02	5.02E-03	2.92E-04
CsI	lime	4.18E-02	4.07E-10	1.76E-10	1.01E-09	3.75E-09	1.62E-03	1.95E-04	2.62E-04	6.71E-04	1.47E-05
CsI	iodine	5.62E+00	1.37E-07	1.35E-08	6.47E-08	2.28E-07	1.52E-01	1.44E-02	1.99E-02	3.37E-02	8.94E-04
CsI	water, deionized	4.98E-04	1.61E-10	2.86E-12	2.60E-11	3.53E-11	3.41E-05	1.75E-06	4.44E-06	5.15E-06	2.01E-07
CsI	rock crushing	4.69E-04	3.54E-12	1.99E-12	5.63E-12	2.03E-11	8.74E-05	1.36E-06	2.29E-06	4.52E-06	2.23E-07
CsI	hydrogen	1.00E+01	2.60E-07	1.53E-08	3.03E-08	7.36E-08	8.53E-02	1.97E-02	1.12E-02	2.42E-02	8.07E-04
MACl	hydrochloric acid	9.89E-01	8.30E-08	6.31E-09	1.21E-07	5.58E-08	7.03E-02	3.24E-03	6.39E-03	1.00E-02	3.87E-04
MACl	methylamine	5.91E+00	6.62E-08	3.84E-08	4.93E-08	4.03E-07	1.37E-01	1.56E-02	3.07E-02	9.33E-02	1.31E-03
MACl	water, deionized	4.98E-04	1.61E-10	2.86E-12	2.60E-11	3.53E-11	3.41E-05	1.75E-06	4.44E-06	5.15E-06	2.01E-07
spiro-OMeTAD	sodium hydroxide	1.49E+00	1.47E-07	1.06E-08	2.89E-08	7.85E-08	1.36E-01	5.05E-03	8.11E-03	1.68E-02	5.99E-04
spiro-OMeTAD	coal tar	1.72E+00	6.85E-09	7.24E-07	5.03E-08	2.96E-07	1.06E-02	3.90E-03	7.21E-03	1.28E-02	1.11E-03
spiro-OMeTAD	sulfur dioxide	4.66E-01	4.35E-09	7.94E-09	1.48E-08	3.93E-07	4.52E-02	5.51E-03	6.30E-02	5.88E-03	2.30E-04
spiro-OMeTAD	phenol	4.59E+00	1.15E-07	9.17E-08	4.80E-08	1.96E-07	1.59E-01	2.03E-02	1.70E-02	3.24E-02	9.14E-04
spiro-OMeTAD	dimethyl sulfate	2.63E+00	2.95E-08	8.78E-09	7.58E-08	2.06E-07	1.35E-01	9.55E-03	2.17E-02	2.31E-02	1.03E-03
spiro-OMeTAD	potassium iodide	5.01E-01	6.75E-09	3.13E-09	2.12E-08	4.80E-08	2.06E-02	2.66E-03	3.09E-03	7.49E-04	8.28E-03
spiro-OMeTAD	sodium chlorate	4.79E+00	3.25E-08	1.02E-08	4.73E-08	2.60E-07	4.46E-01	1.46E-02	2.38E-02	4.97E-02	1.91E-03
spiro-OMeTAD	methanol	9.53E-01	5.50E-08	2.95E-09	2.23E-08	1.18E-08	1.22E-02	5.19E-03	2.20E-03	5.24E-03	1.13E-04
spiro-OMeTAD	hydrochloric acid	9.89E-01	8.30E-08	6.31E-09	1.21E-07	5.58E-08	7.03E-02	3.24E-03	6.39E-03	1.00E-02	3.87E-04
spiro-OMeTAD	hydrogen	1.00E+01	2.60E-07	1.53E-08	3.03E-08	7.36E-08	8.53E-02	1.97E-02	1.12E-02	2.42E-02	8.07E-04
spiro-OMeTAD	sodium methoxide	3.94E+00	1.41E-07	1.31E-08	4.92E-08	2.08E-07	2.93E-01	1.25E-02	1.63E-02	3.36E-02	1.31E-03
spiro-OMeTAD	monochlorobenzene	3.21E+00	1.60E-07	1.81E-07	9.28E-08	1.85E-07	1.42E-01	2.23E-02	1.53E-02	2.88E-02	9.16E-04
spiro-OMeTAD	nitric acid	2.01E+00	1.08E-08	2.32E-09	9.32E-09	6.64E-08	1.26E-02	2.63E-03	1.05E-02	3.89E-02	2.57E-04

spiro-OMeTAD	sulfuric acid	1.81E-01	2.33E-09	3.37E-09	4.71E-08	6.04E-08	1.35E-02	1.94E-03	1.14E-02	5.02E-03	2.92E-04
spiro-OMeTAD	bromine	5.58E+00	1.37E-07	1.35E-08	3.80E-08	2.27E-07	1.53E-01	1.41E-02	2.01E-02	3.25E-02	9.00E-04
spiro-OMeTAD	ethylene glycol	2.98E+00	5.22E-08	7.16E-09	3.75E-08	1.12E-07	1.49E-01	1.10E-02	1.11E-02	2.32E-02	7.70E-04
spiro-OMeTAD	aniline	5.93E+00	9.47E-08	1.78E-07	1.67E-07	2.95E-07	1.55E-01	1.96E-02	3.48E-02	6.35E-02	1.36E-03
spiro-OMeTAD	sodium nitrite	3.56E+00	1.22E-07	2.12E-08	4.58E-08	1.72E-07	2.64E-01	1.21E-02	1.85E-02	4.53E-02	1.10E-03
spiro-OMeTAD	magnesium	2.63E+01	2.21E-07	1.26E-06	2.59E-07	2.06E-05	7.98E-01	6.99E-02	8.43E-02	2.29E-01	5.38E-03
cholorobenzene	monochlorobenzene	3.21E+00	1.60E-07	1.81E-07	9.28E-08	1.85E-07	1.42E-01	2.23E-02	1.53E-02	2.88E-02	9.16E-04
Cu	copper	1.21E+00	1.25E-08	9.78E-09	2.05E-07	1.96E-07	1.16E-01	1.96E-02	2.42E-02	9.93E-02	1.03E-02
EVA	ethylene vinyl acetate copolymer	3.44E+00	8.63E-08	9.50E-09	4.28E-08	1.31E-07	1.41E-01	1.44E-02	1.21E-02	2.52E-02	7.45E-04
Heat	heat	3.89E-02	6.62E-10	6.38E-11	9.32E-11	1.76E-10	1.09E-04	9.82E-05	3.54E-05	1.41E-04	6.50E-07
Electricity (US-NPCC)	electricity, medium voltage	2.30E-01	2.72E-09	5.22E-10	1.18E-09	3.12E-09	3.17E-01	5.51E-04	2.92E-04	9.45E-04	1.21E-05
Electricity (SE)	electricity, medium voltage	2.96E-02	7.52E-10	3.43E-10	8.69E-10	2.47E-09	3.05E-01	9.83E-05	1.43E-04	3.91E-04	9.15E-06
Cooling	cooling energy	2.30E-01	2.72E-09	5.22E-10	1.18E-09	3.12E-09	3.17E-01	5.51E-04	2.92E-04	9.45E-04	1.21E-05

Recycling of 1m^2 Perovskite Layer

DI water	water, deionized	4.98E-04	1.61E-10	2.86E-12	2.60E-11	3.53E-11	3.41E-05	1.75E-06	4.44E-06	5.15E-06	2.01E-07
NaI	sodium	7.27E+00	6.30E-08	1.28E-08	8.31E-08	3.25E-07	1.15E+00	2.18E-02	3.62E-02	7.15E-02	3.22E-03
NaI	iodine	5.62E+00	1.37E-07	1.35E-08	6.47E-08	2.28E-07	1.52E-01	1.44E-02	1.99E-02	3.37E-02	8.94E-04
NaOAc	acetic acid	3.30E+00	6.13E-08	6.92E-09	9.30E-08	1.59E-07	2.26E-01	1.24E-02	1.30E-02	2.57E-02	9.27E-04
NaOAc	sodium hydroxide	1.49E+00	1.47E-07	1.06E-08	2.89E-08	7.85E-08	1.36E-01	5.05E-03	8.11E-03	1.68E-02	5.99E-04
H_3PO_2	phosphorus, white, liquid	1.33E+01	1.26E-07	2.99E-08	3.96E-07	7.14E-07	2.18E+00	5.85E-02	6.85E-02	1.54E-01	6.88E-03
H_3PO_2	barium hydroxide	3.13E+00	2.58E-08	1.19E-07	2.55E-06	2.34E-07	2.13E-01	1.20E-02	1.96E-02	3.88E-02	1.01E-03
H_3PO_2	sulfuric acid	1.81E-01	2.33E-09	3.37E-09	4.71E-08	6.04E-08	1.35E-02	1.94E-03	1.14E-02	5.02E-03	2.92E-04
H_3PO_2	diethyl ether	7.14E+00	3.56E-07	4.02E-08	2.46E-07	4.40E-07	4.07E-01	3.60E-02	5.54E-02	7.11E-02	3.38E-03

Recycling of 1m^2 Spiro Layer

Ehtyl Acetate	ethyl acetate	4.15E+00	8.25E-08	1.10E-08	8.32E-08	2.12E-07	1.93E-01	1.85E-02	1.87E-02	3.56E-02	1.36E-03
Ethanol	ethanol	8.58E-01	5.65E-08	1.62E-08	2.81E-07	7.96E-08	1.30E-02	3.78E-03	1.08E-02	4.46E-02	2.49E-04
MAI	1-butanol	4.84E+00	9.66E-08	1.09E-08	4.74E-08	2.00E-07	2.70E-01	2.00E-02	2.05E-02	3.91E-02	1.50E-03

MAI	hydrogen sulfide	1.46E+00	1.72E-08	1.84E-08	1.10E-08	1.92E-07	3.80E-02	5.30E-03	2.88E-02	8.61E-03	2.72E-04
MAI	iodine	5.62E+00	1.37E-07	1.35E-08	6.47E-08	2.28E-07	1.52E-01	1.44E-02	1.99E-02	3.37E-02	8.94E-04
MAI	methylamine	5.91E+00	6.62E-08	3.84E-08	4.93E-08	4.03E-07	1.37E-01	1.56E-02	3.07E-02	9.33E-02	1.31E-03
MAI	ethanol	8.58E-01	5.65E-08	1.62E-08	2.81E-07	7.96E-08	1.30E-02	3.78E-03	1.08E-02	4.46E-02	2.49E-04
Celite	silica sand	4.32E-02	3.57E-10	1.79E-10	4.89E-10	5.67E-09	6.54E-04	2.43E-04	3.21E-04	7.73E-04	8.90E-06

1m^2 Silicon PV Module

Silicon PV	silicon, single crystal	1.51E+02	1.83E-06	8.61E-07	1.22E-06	6.93E-06	1.51E+01	4.33E-01	6.82E-01	1.42E+00	6.02E-02
Silicon PV	flat glass, uncoated	1.06E+00	1.14E-08	1.63E-09	7.44E-09	1.12E-07	1.62E-02	5.65E-03	1.04E-02	2.04E-02	1.16E-04
Silicon PV	sodium hydroxide	1.49E+00	1.47E-07	1.06E-08	2.89E-08	7.85E-08	1.36E-01	5.05E-03	8.11E-03	1.68E-02	5.99E-04
Silicon PV	hydrochloric acid	9.89E-01	8.30E-08	6.31E-09	1.21E-07	5.58E-08	7.03E-02	3.24E-03	6.39E-03	1.00E-02	3.87E-04
Silicon PV	acetic acid	3.30E+00	6.13E-08	6.92E-09	9.30E-08	1.59E-07	2.26E-01	1.24E-02	1.30E-02	2.57E-02	9.27E-04
Silicon PV	dipropylene glycol monomethyl ether	6.51E+00	3.41E-07	3.11E-08	4.16E-07	3.14E-07	4.02E-01	2.45E-02	2.80E-02	5.76E-02	2.05E-03
Silicon PV	alkylbenzene sulfonate	1.74E+00	4.62E-08	3.85E-08	6.58E-08	1.42E-07	5.62E-02	8.22E-03	1.29E-02	1.94E-02	4.27E-04
Silicon PV	acrylic binder	2.89E+00	5.01E-08	7.84E-09	1.44E-07	1.52E-07	1.56E-01	1.20E-02	1.32E-02	2.60E-02	8.43E-04
Silicon PV	brass	5.87E+00	5.55E-08	7.39E-08	4.29E-06	9.51E-07	6.90E-01	8.24E-02	4.03E-01	2.93E-01	3.21E-02
Silicon PV	steel	5.11E+00	3.47E-08	1.28E-07	1.24E-07	4.19E-07	3.04E-01	1.70E-02	2.83E-02	5.46E-02	1.64E-03
Silicon PV	wire drawing, steel	3.79E-01	3.30E-09	9.55E-09	7.17E-09	1.64E-08	1.43E-02	8.85E-04	1.26E-03	2.65E-03	1.96E-04
Silicon PV	water, deionised	4.98E-04	1.61E-10	2.86E-12	2.60E-11	3.53E-11	3.41E-05	1.75E-06	4.44E-06	5.15E-06	2.01E-07
Silicon PV	waste, from silicon wafer production	1.37E-01	1.02E-09	1.00E-08	2.66E-09	1.24E-08	2.27E-03	6.24E-04	6.20E-04	1.90E-03	3.73E-05
Silicon PV	treatment, sewage	1.05E-01	4.65E-11	3.11E-10	3.69E-08	5.56E-09	8.78E-05	7.88E-05	8.26E-04	3.71E-03	1.12E-04
Silicon PV	wafer factory	1.06E+05	9.92E-04	3.43E-03	8.64E-03	1.27E-02	5.13E+03	4.90E+02	1.20E+03	2.41E+03	7.10E+01
Silicon PV	metallization paste, front side	3.99E+02	4.21E-06	1.06E-06	1.48E-05	3.04E-05	4.22E+01	3.92E+00	4.52E+00	1.65E+01	1.09E+00
Silicon PV	metallization paste, back side	3.23E+02	3.42E-06	8.60E-07	1.20E-05	2.46E-05	3.41E+01	3.17E+00	3.65E+00	1.33E+01	8.80E-01

Silicon PV	metallization paste, back side, aluminium	1.21E+01	8.28E-08	3.25E-08	1.73E-07	9.77E-07	2.34E-01	4.32E-02	7.94E-02	1.36E-01	3.66E-03
Silicon PV	ammonia	3.23E+00	3.59E-08	4.69E-09	1.50E-08	1.00E-07	1.95E-02	6.87E-03	8.83E-03	1.74E-02	7.38E-04
Silicon PV	phosphorus oxychloride	4.63E+00	1.11E-07	1.55E-08	3.09E-07	2.49E-07	6.26E-01	1.88E-02	2.49E-02	5.44E-02	1.30E-02
Silicon PV	isopropanol	3.37E+00	6.88E-08	8.60E-09	3.52E-08	1.55E-07	9.29E-02	1.51E-02	1.35E-02	2.38E-02	7.23E-04
Silicon PV	hydrochloric acid	9.89E-01	8.30E-08	6.31E-09	1.21E-07	5.58E-08	7.03E-02	3.24E-03	6.39E-03	1.00E-02	3.87E-04
Silicon PV	hydrogen fluoride	1.51E+00	4.95E-08	1.31E-08	1.39E-07	2.42E-07	9.15E-02	9.38E-03	3.58E-02	2.67E-02	1.09E-03
Silicon PV	sodium hydroxide	1.49E+00	1.47E-07	1.06E-08	2.89E-08	7.85E-08	1.36E-01	5.05E-03	8.11E-03	1.68E-02	5.99E-04
Silicon PV	lime	9.95E-01	5.26E-09	7.90E-10	8.05E-09	1.30E-08	6.51E-03	1.73E-03	1.45E-03	3.84E-03	3.12E-05
Silicon PV	refrigerant R134a	1.72E+01	7.83E-04	3.85E-08	2.48E-07	6.38E-07	3.58E-01	3.97E-02	5.43E-02	7.73E-02	1.72E-03
Silicon PV	nitrogen	4.50E-01	2.55E-09	4.90E-10	2.95E-09	2.11E-08	4.29E-02	1.34E-03	2.19E-03	4.59E-03	1.78E-04
Silicon PV	silane	3.04E+01	2.47E-06	2.47E-08	2.19E-07	5.35E-07	1.09E+01	7.70E-02	9.24E-02	1.95E-01	1.06E-02
Silicon PV	tap water	1.28E-03	2.31E-10	2.02E-11	4.20E-11	8.58E-11	1.07E-04	4.22E-06	6.62E-06	1.38E-05	4.31E-07
Silicon PV	photovoltaic cell factory	1.41E+06	9.20E-03	7.11E-02	3.64E-02	1.37E-01	3.40E+04	5.61E+03	6.22E+03	1.74E+04	4.01E+02
Silicon PV	transport, freight lorry	1.96E-01	2.75E-09	1.06E-09	2.78E-09	2.09E-08	2.26E-03	1.56E-03	1.11E-03	4.92E-03	1.54E-05
Silicon PV	transport, freight train	5.24E-02	5.57E-10	5.95E-10	6.22E-10	7.11E-09	1.91E-03	5.64E-04	4.63E-04	1.96E-03	9.82E-06
Silicon PV	wastewater from PV cell production	2.39E+00	1.16E-08	4.61E-09	1.40E-07	9.08E-08	1.00E-01	5.39E-03	8.76E-03	2.97E-02	1.38E-02
Silicon PV	waste, from silicon wafer production, inorganic	3.61E-01	1.64E-09	4.92E-10	3.20E-09	1.21E-08	3.45E-03	1.11E-03	1.16E-03	3.85E-03	1.35E-04
Silicon PV	spent solvent mixture	2.00E+00	9.18E-09	1.27E-09	3.87E-09	1.83E-08	7.47E-03	1.13E-03	1.20E-03	3.88E-03	2.83E-04
Silicon PV	aluminium alloy, AlMg3	7.60E+00	5.12E-08	6.06E-08	1.09E-07	1.13E-06	3.59E-01	2.61E-02	4.54E-02	8.44E-02	2.44E-03
Silicon PV	copper	1.21E+00	1.25E-08	9.78E-09	2.05E-07	1.96E-07	1.16E-01	1.96E-02	2.42E-02	9.93E-02	1.03E-02

Silicon PV	wire drawing, copper	7.11E-01	6.48E-09	5.14E-09	2.38E-07	7.76E-08	8.67E-02	6.40E-03	2.42E-02	1.90E-02	1.91E-03
Silicon PV	diode	2.60E+02	2.62E-06	3.76E-07	2.42E-06	1.43E-05	3.00E+01	8.52E-01	1.44E+00	2.95E+00	1.17E-01
Silicon PV	silicone product	3.71E+00	1.33E-06	5.72E-08	5.01E-08	2.28E-07	1.76E-01	1.33E-02	1.78E-02	3.71E-02	8.85E-04
Silicon PV	tin	1.05E+01	9.24E-08	2.70E-08	4.37E-07	1.48E-06	9.32E-01	9.23E-02	1.28E-01	4.01E-01	2.45E-02
Silicon PV	lead	1.42E+00	3.17E-08	1.29E-08	7.13E-07	1.65E-07	1.10E-01	7.88E-03	3.31E-02	2.29E-02	1.44E-03
Silicon PV	solar glass, low-iron	1.12E+00	1.58E-08	2.08E-09	8.17E-09	1.16E-07	2.43E-02	6.16E-03	1.06E-02	2.18E-02	1.23E-04
Silicon PV	tempering, flat glass	1.79E-01	3.21E-09	2.80E-10	9.56E-10	1.32E-08	2.96E-03	7.89E-04	1.27E-03	2.59E-03	1.46E-05
Silicon PV	glass fibre reinforced plastic	8.96E+00	3.44E-08	5.11E-09	2.19E-08	5.20E-07	2.39E-01	2.31E-02	3.80E-02	6.48E-02	6.59E-04
Silicon PV	polyethylene terephthalate	3.89E+00	1.51E-05	1.16E-08	5.72E-08	1.77E-07	2.05E-01	1.61E-02	1.70E-02	3.14E-02	8.77E-04
Silicon PV	polyethylene, high density	3.10E+00	9.25E-08	1.01E-08	3.06E-08	1.17E-07	1.25E-01	1.46E-02	1.09E-02	2.32E-02	6.19E-04
Silicon PV	ethylvinylacetate	3.98E+00	1.03E-07	1.20E-08	4.85E-08	1.58E-07	2.76E-01	1.71E-02	1.47E-02	3.22E-02	9.51E-04
Silicon PV	polyvinylfluoride	2.07E+01	2.17E-07	3.69E-08	2.85E-07	7.97E-07	1.53E+00	6.85E-02	1.05E-01	2.10E-01	5.65E-03
Silicon PV	tap water	1.28E-03	2.31E-10	2.02E-11	4.20E-11	8.58E-11	1.07E-04	4.22E-06	6.62E-06	1.38E-05	4.31E-07
Silicon PV	hydrogen fluoride	1.51E+00	4.95E-08	1.31E-08	1.39E-07	2.42E-07	9.15E-02	9.38E-03	3.58E-02	2.67E-02	1.09E-03
Silicon PV	1-propanol	7.61E+00	9.34E-08	1.49E-08	5.92E-08	3.28E-07	3.33E-01	5.50E-02	4.20E-02	7.78E-02	3.38E-03
Silicon PV	isopropanol	3.37E+00	6.88E-08	8.60E-09	3.52E-08	1.55E-07	9.29E-02	1.51E-02	1.35E-02	2.38E-02	7.23E-04
Silicon PV	potassium hydroxide	4.74E+00	5.60E-08	2.96E-08	8.91E-08	2.75E-07	4.44E-01	1.61E-02	2.55E-02	5.22E-02	1.88E-03
Silicon PV	soap	4.62E+00	4.68E-08	1.49E-08	1.09E-07	3.26E-07	9.60E-02	1.20E-02	2.00E-02	7.28E-02	2.96E-02
Silicon PV	corrugated board box	1.34E+00	2.80E-08	4.26E-09	2.78E-08	1.24E-07	7.13E-02	5.86E-03	6.45E-03	1.88E-02	6.03E-04
Silicon PV	EUR-flat pallet	8.87E+00	1.06E-07	1.66E-07	1.39E-07	1.28E-06	3.38E-01	6.12E-02	5.75E-02	1.75E-01	2.22E-03

Silicon PV	photovoltaic panel factory	3.42E+05	2.92E-03	7.56E-03	8.32E-03	5.29E-02	1.10E+04	1.56E+03	3.30E+03	1.13E+04	9.34E+01
Silicon PV	municipal solid waste	5.19E-01	4.93E-10	5.96E-10	6.25E-09	2.99E-09	4.89E-04	3.67E-04	3.05E-04	1.41E-03	4.59E-05
Silicon PV	waste polyvinylfluoride	2.31E+00	2.80E-09	8.52E-10	3.04E-08	8.33E-09	3.78E-03	9.55E-04	9.05E-04	3.34E-03	5.81E-05
Silicon PV	waste plastic, mixture	2.38E+00	8.78E-10	5.46E-10	8.02E-09	2.80E-09	8.49E-04	6.61E-04	5.43E-04	2.63E-03	7.48E-06
Silicon PV	waste mineral oil	2.85E+00	5.86E-10	7.03E-10	3.45E-09	1.13E-08	6.12E-04	2.63E-04	2.60E-04	1.17E-03	1.85E-04
Silicon PV	treatment, sewage	1.05E-01	4.65E-11	3.11E-10	3.69E-08	5.56E-09	8.78E-05	7.88E-05	8.26E-04	3.71E-03	1.12E-04
**Recycling of 1m^2 Silicon PV Module**											
Silicon Recycling	electricity, medium voltage (ENTSO-E)	3.11E-01	5.52E-09	6.50E-10	3.22E-09	5.62E-09	2.06E-01	7.39E-04	1.51E-03	2.21E-03	2.95E-04
Silicon Recycling	diesel	1.00E-01	1.53E-09	3.92E-10	5.69E-10	2.57E-08	5.87E-04	1.37E-03	9.04E-04	4.59E-03	2.93E-06
Silicon Recycling	waste plastic plaster	1.40E-02	3.40E-10	6.25E-11	6.37E-10	1.63E-09	3.72E-04	1.16E-04	6.59E-05	3.01E-04	7.35E-07
Silicon Recycling	natural gas	7.56E-02	2.12E-09	1.25E-10	1.57E-10	5.08E-10	2.00E-04	2.69E-04	1.45E-04	6.91E-04	1.20E-06
Silicon Recycling	heavy fuel oil	9.35E-02	1.76E-09	8.78E-11	6.91E-10	1.58E-08	4.18E-04	3.82E-04	1.23E-03	6.67E-04	9.54E-07
Silicon Recycling	silica sand	2.76E-02	2.75E-10	9.90E-11	3.12E-10	2.80E-09	5.61E-04	1.55E-04	1.99E-04	4.92E-04	7.59E-06
Silicon Recycling	soda, powder	3.47E-01	3.53E-09	1.82E-09	1.34E-08	3.83E-08	2.43E-02	1.36E-03	4.88E-03	1.40E-02	1.96E-04
Silicon Recycling	limestone	2.35E-03	3.33E-11	1.45E-11	2.81E-11	8.15E-10	6.49E-05	5.70E-05	5.18E-05	2.60E-04	1.67E-07
Silicon Recycling	aluminium	2.23E+01	1.02E-07	3.68E-08	2.67E-07	1.85E-06	1.68E-01	7.68E-02	1.44E-01	2.53E-01	6.37E-03

Supplementary Table 12. Characterization factors for production extracted from the Eco-invent database V3.10 (Part II)

Method	EF v3.0	EF v3.0	EF v3.0	EF v3.0	EF v3.0	Cumulative Energy Demand (CED)										
Category	Eutrophication: marine	Ecotoxicity: freshwater	Land use	Water use	Metals/ minerals	Renewable	Non-ren	Non-ren biomass	Fossil	Nuclear	Ren biomass	Geothermal	Solar	Water	Wind	Total
Indicator	Fraction of nutrients reaching marine end compartment	Comparative toxic unit for ecosystems	Soil quality index	User deprivation potential	Abiotic depletion potential	Energy content (HHV)										
Materials	kg N-Eq	CTUe	dimensionless	m^3 world Eq deprived	kg Sb-Eq	MJ-Eq	MJ-Eq	MJ-Eq	MJ-Eq	MJ-Eq	MJ-Eq	MJ-Eq	MJ-Eq	MJ-Eq	MJ-Eq	MJ-Eq
**$1m^2$ Perovskite module**																
hindium	2.58E-01	3.55E+04	8.29E+02	1.46E+02	6.40E-02	2.74E+02	1.80E+03	3.76E-02	1.41E+03	3.92E+02	3.31E+01	1.47E+01	4.43E-02	1.52E+02	7.37E+01	2.07E+03
tin	2.97E-02	3.85E+03	8.95E+01	7.59E+00	2.69E-02	1.89E+01	1.42E+02	5.09E-03	1.27E+02	1.49E+01	7.70E+00	8.05E-01	4.38E-03	7.59E+00	2.76E+00	1.61E+02
glass cullet, sorted	3.54E-05	7.18E+01	1.51E-01	5.72E-03	4.47E-08	6.80E-03	1.75E-01	1.48E-04	1.68E-01	6.98E-03	1.26E-03	4.60E-04	1.35E-05	3.71E-03	1.36E-03	1.82E-01
titanium dioxide	7.51E-03	1.72E+03	4.71E+01	3.78E+00	8.61E-05	5.76E+00	8.01E+01	5.60E-03	7.62E+01	3.93E+00	2.49E+00	2.60E-01	7.82E-03	2.18E+00	8.21E-01	8.59E+01
argon, liquid	2.44E-03	6.81E+01	5.16E+00	2.75E+00	2.67E-06	4.15E+00	3.52E+01	2.78E-04	2.93E+01	5.91E+00	5.42E-01	3.20E-01	1.53E-04	2.16E+00	1.13E+00	3.94E+01
oxygen, liquid	1.12E-03	3.07E+01	2.13E+00	1.10E+00	1.03E-06	1.46E+00	1.44E+01	1.59E-04	1.28E+01	1.67E+00	1.82E-01	1.22E-01	6.37E-05	8.15E-01	3.38E-01	1.59E+01
tin dioxide	2.49E-02	3.20E+03	7.57E+01	6.38E+00	2.23 E-02	1.58E+01	1.21E+02	5.00E-03	1.09E+02	1.25E+01	6.47E+00	6.74E-01	3.94E-03	6.37E+00	2.31E+00	1.37E+02
water, deionized	4.90E-07	1.61E+00	2.08E-03	8.64E-04	2.08E-08	5.45E-04	6.25E-03	4.99E-07	5.71E-03	5.40E-04	1.16E-04	3.17E-05	1.80E-05	2.78E-04	1.01E-04	6.80E-03
lead	2.11E-03	3.23E+02	1.35E+01	1.12E+00	1.32E-03	2.15E+00	1.77E+01	1.86E-03	1.60E+01	1.69E+00	5.86E-01	1.17E-01	1.27E-03	1.07E+00	3.81E-01	1.99E+01

iodine	3.24E-03	2.87E+02	7.57E+00	1.44E+02	2.17E-05	2.23E+00	8.00E+01	1.50E-03	7.74E+01	2.53E+00	7.24E-01	1.04E-01	1.10E-02	8.63E-01	5.31E-01	8.22E+01
potassium hydroxide	5.12E-03	1.54E+03	1.94E+01	1.28E+00	8.83E-05	6.22E+00	6.37E+01	4.80E-03	5.65E+01	7.15E+00	1.41E+00	4.00E-01	6.93E-03	2.94E+00	1.47E+00	6.99E+01
nitric acid	9.33E-04	2.08E+01	2.72E+00	6.84E-01	1.01E-05	4.48E-01	1.18E+01	1.53E-04	1.16E+01	2.02E-01	2.76E-01	1.01E-02	4.93E-03	1.24E-01	3.33E-02	1.23E+01
water, deionized	4.90E-07	1.61E+00	2.08E-03	8.64E-04	2.08E-08	5.45E-04	6.25E-03	4.99E-07	5.71E-03	5.40E-04	1.16E-04	3.17E-05	1.80E-05	2.78E-04	1.01E-04	6.80E-03
iodine	3.24E-03	2.87E+02	7.57E+00	1.44E+02	2.17E-05	2.23E+00	8.00E+01	1.50E-03	7.74E+01	2.53E+00	7.24E-01	1.04E-01	1.10E-02	8.63E-01	5.31E-01	8.22E+01
hydrogen sulfide	8.13E-04	3.77E+01	2.67E+00	8.64E-01	9.83E-06	6.02E-01	2.26E+01	1.07E-03	2.20E+01	5.94E-01	1.60E-01	3.73E-02	3.30E-04	2.93E-01	1.11E-01	2.32E+01
trichloroborane	4.42E-03	1.54E+02	1.35E+01	1.36E+00	9.26E-04	4.66E+00	4.74E+01	2.83E-03	4.20E+01	5.40E+00	8.74E-01	3.46E-01	4.60E-03	2.37E+00	1.07E+00	5.21E+01
sodium	7.17E-03	2.35E+02	1.88E+01	2.18E+00	7.21E-05	1.28E+01	1.05E+02	2.92E-03	8.59E+01	1.91E+01	1.99E+00	8.98E-01	2.91E-03	6.34E+00	3.56E+00	1.18E+02
acetic acid	2.50E-03	1.11E+02	7.92E+00	1.81E+00	1.44E-05	2.93E+00	6.71E+01	1.22E-03	6.35E+01	3.66E+00	5.88E-01	1.99E-01	1.97E-03	1.44E+00	7.03E-01	7.01E+01
ammonia, anhydrous	1.68E-03	5.28E+01	6.46E+00	2.46E+00	9.60E-06	1.01E+00	4.03E+01	3.27E-04	4.00E+01	3.01E-01	7.22E-01	1.67E-02	9.98E-03	2.15E-01	4.75E-02	4.13E+01
ethanol	2.11E-03	9.65E+01	7.43E+00	5.71E-01	1.63E-05	1.00E+00	5.70E+01	1.34E-03	5.62E+01	7.82E-01	4.29E-01	4.20E-02	1.71E-03	3.86E-01	1.42E-01	5.80E+01
N, N-dimethylformamide	4.02E-03	2.01E+02	1.27E+01	2.27E+00	2.50E-05	4.05E+00	1.03E+02	2.00E-03	9.86E+01	4.62E+00	9.28E-01	2.64E-01	5.43E-03	1.96E+00	8.98E-01	1.07E+02
dimethyl sulfoxide	1.84E-03	2.21E+03	6.67E+00	1.09E+00	1.64E-05	1.89E+00	6.01E+01	1.50E-03	5.79E+01	2.22E+00	4.58E-01	1.15E-01	7.73E-04	8.92E-01	4.26E-01	6.20E+01
pollucite ore	9.14E-05	1.45E-02	4.49E-02	7.86E-03	2.37E-07	4.56E-02	5.02E-01	1.56E-02	4.62E-01	8.80E-04	4.05E-02	4.26E-05	1.35E-05	2.44E-02	4.65E-03	5.48E-01
sulfuric acid	4.19E-04	3.94E+01	2.32E+00	5.59E-01	6.48E-05	4.17E-01	2.51E+00	8.65E-04	2.31E+00	2.00E-01	1.27E-01	9.69E-03	4.21E-03	2.40E-01	3.69E-02	2.93E+00
lime	5.73E-05	4.67E+00	2.00E-01	4.32E-02	4.38E-06	9.45E-02	5.44E-01	7.86E-05	5.19E-01	2.45E-02	1.39E-02	2.00E-03	1.08E-04	7.35E-02	5.09E-03	6.38E-01

iodine	3.24E-03	2.87E+02	7.57E+00	1.44E+02	2.17E-05	2.23E+00	8.00E+01	1.50E-03	7.74E+01	2.53E+00	7.24E-01	1.04E-01	1.10E-02	8.63E-01	5.31E-01	8.22E+01
water, deionized	4.90E-07	1.61E+00	2.08E-03	8.64E-04	2.08E-08	5.45E-04	6.25E-03	4.99E-07	5.71E-03	5.40E-04	1.16E-04	3.17E-05	1.80E-05	2.78E-04	1.01E-04	6.80E-03
rock crushing	4.54E-07	1.33E-02	1.05E-03	1.19E-04	5.72E-10	9.29E-04	6.92E-03	5.56E-08	5.46E-03	1.46E-03	1.24E-04	6.72E-05	3.56E-08	4.66E-04	2.71E-04	7.85E-03
hydrogen	2.38E-03	1.12E+02	5.72E+00	1.48E+00	2.21E-05	1.34E+00	1.84E+02	1.15E-03	1.82E+02	1.31E+00	3.66E-01	8.25E-02	1.36E-03	6.59E-01	2.26E-01	1.85E+02
hydrochloric acid	9.51E-04	4.50E+01	3.34E+00	5.06E-01	2.75E-05	1.11E+00	1.37E+01	7.36E-04	1.26E+01	1.10E+00	2.79E-01	7.03E-02	1.21E-03	5.46E-01	2.14E-01	1.48E+01
methylamine	3.75E-03	2.25E+02	1.18E+01	2.51E+00	1.93E-05	2.57E+00	9.20E+01	1.63E-03	8.98E+01	2.13E+00	8.63E-01	1.43E-01	5.16E-03	1.13E+00	4.33E-01	9.46E+01
water, deionized	4.90E-07	1.61E+00	2.08E-03	8.64E-04	2.08E-08	5.45E-04	6.25E-03	4.99E-07	5.71E-03	5.40E-04	1.16E-04	3.17E-05	1.80E-05	2.78E-04	1.01E-04	6.80E-03
sodium hydroxide	1.67E-03	5.83E+01	4.60E+00	7.05E-01	2.70E-05	1.90E+00	1.95E+01	1.51E-03	1.73E+01	2.14E+00	3.52E-01	1.38E-01	1.05E-03	9.89E-01	4.16E-01	2.13E+01
coal tar	1.28E-03	4.42E+02	8.35E+00	2.10E-01	5.70E-07	6.73E-01	4.48E+01	3.34E-04	4.46E+01	1.73E-01	5.16E-01	6.56E-03	2.78E-04	1.04E-01	4.57E-02	4.55E+01
sulfur dioxide	5.56E-04	3.51E+01	1.92E+00	2.06E-01	4.13E-05	6.39E-01	6.38E+00	3.69E-04	5.68E+00	6.97E-01	1.32E-01	4.01E-02	1.33E-04	3.31E-01	1.36E-01	7.02E+00
phenol	3.07E-03	1.77E+02	1.21E+01	7.45E-01	3.51E-05	2.38E+00	1.11E+02	2.54E-03	1.09E+02	2.60E+00	7.15E-01	1.26E-01	4.17E-03	1.05E+00	4.83E-01	1.14E+02
dimethyl sulfate	2.17E-03	1.48E+02	8.80E+00	1.33E+00	8.41E-05	2.38E+00	4.37E+01	1.82E-03	4.16E+01	2.10E+00	6.03E-01	1.39E-01	4.69E-03	1.21E+00	4.22E-01	4.61E+01
potassium iodide	1.55E-04	1.47E+03	6.18E+00	3.14E-01	2.77E-05	4.14E-01	7.18E+00	2.91E-03	6.85E+00	3.30E-01	1.50E-01	1.78E-02	2.25E-03	1.81E-01	6.34E-02	7.60E+00
sodium chlorate	4.95E-03	2.11E+02	1.08E+01	4.83E+00	2.71E-05	6.09E+00	6.02E+01	1.63E-03	5.33E+01	6.91E+00	8.90E-01	4.97E-01	6.24E-04	3.30E+00	1.39E+00	6.63E+01
methanol	5.10E-04	1.84E+01	1.01E+00	1.02E-01	1.77E-06	2.02E-01	4.16E+01	5.26E-04	4.14E+01	1.74E-01	3.17E-02	2.06E-02	1.25E-04	1.20E-01	2.89E-02	4.18E+01
hydrochloric acid	9.51E-04	4.50E+01	3.34E+00	5.06E-01	2.75E-05	1.11E+00	1.37E+01	7.36E-04	1.26E+01	1.10E+00	2.79E-01	7.03E-02	1.21E-03	5.46E-01	2.14E-01	1.48E+01

hydrogen	2.38E-03	1.12E+02	5.72E+00	1.48E+00	2.21E-05	1.34E+00	1.84E+02	1.15E-03	1.82E+02	1.31E+00	3.66E-01	8.25E-02	1.36E-03	6.59E-01	2.26E-01	1.85E+02
sodium methoxide	3.31E-03	1.55E+02	9.89E+00	1.27E+00	3.39E-05	3.91E+00	6.28E+01	2.21E-03	5.81E+01	4.67E+00	7.56E-01	2.65E-01	1.70E-03	1.98E+00	9.10E-01	6.67E+01
monochlorobenzene	2.76E-03	5.03E+02	1.10E+01	7.80E-01	3.94E-05	2.33E+00	8.28E+01	2.29E-03	8.06E+01	2.24E+00	6.86E-01	1.37E-01	2.37E-03	1.07E+00	4.30E-01	8.51E+01
nitric acid	9.33E-04	2.08E+01	2.72E+00	6.84E-01	1.01E-05	4.48E-01	1.18E+01	1.53E-04	1.16E+01	2.02E-01	2.76E-01	1.01E-02	4.93E-03	1.24E-01	3.33E-02	1.23E+01
sulfuric acid	4.19E-04	3.94E+01	2.32E+00	5.59E-01	6.48E-05	4.17E-01	2.51E+00	8.65E-04	2.31E+00	2.00E-01	1.27E-01	9.69E-03	4.21E-03	2.40E-01	3.69E-02	2.93E+00
bromine	3.12E-03	1.07E+02	7.32E+00	1.92E+01	2.41E-05	2.25E+00	7.95E+01	1.03E-03	7.69E+01	2.54E+00	7.33E-01	1.04E-01	1.15E-02	8.67E-01	5.35E-01	8.17E+01
ethylene glycol	2.26E-03	9.41E+01	7.49E+00	1.06E+00	2.12E-05	2.25E+00	5.94E+01	1.37E-03	5.71E+01	2.31E+00	4.85E-01	1.56E-01	1.26E-03	1.15E+00	4.59E-01	6.16E+01
aniline	4.79E-03	5.31E+02	1.53E+01	1.82E+00	1.07E-04	2.87E+00	1.11E+02	4.25E-03	1.08E+02	2.41E+00	8.82E-01	1.46E-01	8.06E-03	1.38E+00	4.59E-01	1.14E+02
sodium nitrite	5.58E-03	1.17E+02	9.14E+00	2.16E+00	3.55E-05	3.48E+00	4.82E+01	2.68E-03	4.39E+01	4.29E+00	7.45E-01	2.29E-01	6.97E-03	1.69E+00	8.11E-01	5.17E+01
magnesium	2.16E-02	1.13E+03	4.74E+01	2.38E+00	4.83E-05	1.42E+01	2.60E+02	1.06E-02	2.47E+02	1.29E+01	2.66E+00	5.83E-01	1.58E+00	6.50E+00	2.93E+00	2.74E+02
monochlorobenzene	2.76E-03	5.03E+02	1.10E+01	7.80E-01	3.94E-05	2.33E+00	8.28E+01	2.29E-03	8.06E+01	2.24E+00	6.86E-01	1.37E-01	2.37E-03	1.07E+00	4.30E-01	8.51E+01
copper	6.78E-03	1.36E+03	6.50E+01	1.19E+00	3.08E-03	4.17E+00	1.59E+01	1.23E-03	1.43E+01	1.63E+00	4.54E-01	1.09E-01	1.55E-03	3.27E+00	3.31E-01	2.01E+01
ethylene vinyl acetate copolymer	2.43E-03	1.13E+02	9.42E+00	9.67E-01	2.44E-05	2.24E+00	8.87E+01	1.22E-03	8.64E+01	2.23E+00	6.44E-01	1.32E-01	2.34E-03	1.03E+00	4.31E-01	9.09E+01
heat	1.31E-05	3.10E-01	1.32E-02	2.42E-03	2.32E-08	1.39E-03	6.84E-01	2.94E-06	6.82E-01	1.61E-03	3.35E-04	6.95E-05	2.55E-05	7.32E-04	2.26E-04	6.85E-01
electricity, medium voltage (US-NPCC)	9.58E-05	5.40E+00	1.50E+00	2.32E-01	5.94E-07	1.17E+00	8.50E+00	3.59E-05	3.99E+00	4.51E+00	3.00E-01	8.03E-04	2.05E-05	7.03E-01	1.68E-01	9.67E+00
electricity, medium voltage (SE)	4.25E-05	1.98E+00	8.94E-01	2.30E-01	5.90E-07	2.86E+00	4.20E+00	2.08E-05	1.74E-01	4.02E+00	2.05E-01	4.73E-04	1.90E-05	1.95E+00	7.04E-01	7.05E+00

cooling energy	3.27E-05	1.57E+00	7.76E-02	3.12E-02	7.50E-07	3.53E-02	1.06E+00	7.99E-06	1.02E+00	3.86E-02	4.73E-03	2.05E-03	3.64E-03	1.82E-02	6.70E-03	1.10E+00

Recycling of 1m^2 Perovskite Layer

water, deionized	4.90E-07	1.61E+00	2.08E-03	8.64E-04	2.08E-08	5.45E-04	6.25E-03	4.99E-07	5.71E-03	5.40E-04	1.16E-04	3.17E-05	1.80E-05	2.78E-04	1.01E-04	6.80E-03
sodium	7.17E-03	2.35E+02	1.88E+01	2.18E+00	7.21E-05	1.28E+01	1.05E+02	2.92E-03	8.59E+01	1.91E+01	1.99E+00	8.98E-01	2.91E-03	6.34E+00	3.56E+00	1.18E+02
iodine	3.24E-03	2.87E+02	7.57E+00	1.44E+02	2.17E-05	2.23E+00	8.00E+01	1.50E-03	7.74E+01	2.53E+00	7.24E-01	1.04E-01	1.10E-02	8.63E-01	5.31E-01	8.22E+01
acetic acid	2.50E-03	1.11E+02	7.92E+00	1.81E+00	1.44E-05	2.93E+00	6.71E+01	1.22E-03	6.35E+01	3.66E+00	5.88E-01	1.99E-01	1.97E-03	1.44E+00	7.03E-01	7.01E+01
sodium hydroxide	1.67E-03	5.83E+01	4.60E+00	7.05E-01	2.70E-05	1.90E+00	1.95E+01	1.51E-03	1.73E+01	2.14E+00	3.52E-01	1.38E-01	1.05E-03	9.89E-01	4.16E-01	2.13E+01
phosphorus, white, liquid	1.60E-02	3.90E+02	1.20E+02	4.49E+00	5.36E-05	2.41E+01	2.13E+02	1.18E-01	1.82E+02	3.09E+01	6.48E+00	1.43E+00	2.45E-01	1.02E+01	5.79E+00	2.37E+02
barium hydroxide	3.65E-03	2.16E+03	1.03E+01	2.83E+00	4.40E-05	2.91E+00	3.92E+01	3.99E-03	3.58E+01	3.42E+00	6.81E-01	1.80E-01	1.32E-03	1.44E+00	6.15E-01	4.21E+01
sulfuric acid	4.19E-04	3.94E+01	2.32E+00	5.59E-01	6.48E-05	4.17E-01	2.51E+00	8.65E-04	2.31E+00	2.00E-01	1.27E-01	9.69E-03	4.21E-03	2.40E-01	3.69E-02	2.93E+00
diethyl ether	6.76E-03	4.95E+04	2.62E+01	3.82E+00	2.09E-04	6.60E+00	1.52E+02	5.89E-03	1.45E+02	6.49E+00	1.85E+00	3.51E-01	1.68E-02	3.09E+00	1.29E+00	1.58E+02

Recycling of 1m^2 Spiro Layer

ethyl acetate	3.40E-03	1.48E+02	1.32E+01	1.82E+00	2.70E-05	3.16E+00	8.72E+01	2.27E-03	8.41E+01	3.14E+00	1.13E+00	1.64E-01	3.51E-03	1.27E+00	5.93E-01	9.04E+01
ethanol	4.58E-03	3.06E+02	8.91E+01	6.07E+00	4.33E-06	4.22E+01	5.98E+00	1.28E-01	5.65E+00	1.97E-01	4.20E+01	1.27E-02	7.17E-03	1.11E-01	3.60E-02	4.82E+01
1-butanol	3.77E-03	1.57E+02	1.36E+01	1.23E+00	2.23E-05	3.84E+00	1.03E+02	1.59E-03	9.85E+01	4.45E+00	1.23E+00	2.09E-01	3.34E-03	1.57E+00	8.22E-01	1.07E+02
hydrogen sulfide	8.13E-04	3.77E+01	2.67E+00	8.64E-01	9.83E-06	6.02E-01	2.26E+01	1.07E-03	2.20E+01	5.94E-01	1.60E-01	3.73E-02	3.30E-04	2.93E-01	1.11E-01	2.32E+01
iodine	3.24E-03	2.87E+02	7.57E+00	1.44E+02	2.17E-05	2.23E+00	8.00E+01	1.50E-03	7.74E+01	2.53E+00	7.24E-01	1.04E-01	1.10E-02	8.63E-01	5.31E-01	8.22E+01

methylamine	3.75E-03	2.25E+02	1.18E+01	2.51E+00	1.93E-05	2.57E+00	9.20E+01	1.63E-03	8.98E+01	2.13E+00	8.63E-01	1.43E-01	5.16E-03	1.13E+00	4.33E-01	9.46E+01
ethanol	4.58E-03	3.06E+02	8.91E+01	6.07E+00	4.33E-06	4.22E+01	5.98E+00	1.28E-01	5.65E+00	1.97E-01	4.20E+01	1.27E-02	7.17E-03	1.11E-01	3.60E-02	4.82E+01
silica sand	7.13E-05	1.23E+00	1.11E+00	5.03E-03	9.52E-08	3.28E-02	5.02E-01	1.53E-04	4.91E-01	1.04E-02	2.54E-02	5.64E-04	1.42E-04	4.75E-03	1.92E-03	5.35E-01

1m^2 Silicon PV Module

silicon, single crystal	1.40E-01	4.23E+03	3.76E+02	2.94E+02	1.95E-04	2.01E+03	2.29E-02	1.77E+03	2.43E+02	4.55E+02	4.99E+01	1.45E+01	2.53E-02	3.33E+02	5.73E+01	2.46E+03
flat glass, uncoated	1.69E-03	2.66E+01	3.35E+00	2.30E-01	9.61E-06	1.20E+01	3.83E-04	1.17E+01	2.53E-01	5.16E-01	2.93E-01	1.58E-02	4.02E-04	1.58E-01	4.89E-02	1.25E+01
sodium hydroxide	1.67E-03	5.83E+01	4.60E+00	7.05E-01	2.70E-05	1.95E+01	1.51E-03	1.73E+01	2.14E+00	1.90E+00	3.52E-01	1.38E-01	1.05E-03	9.89E-01	4.16E-01	2.13E+01
hydrochloric acid	9.51E-04	4.50E+01	3.34E+00	5.06E-01	2.75E-05	1.37E+01	7.36E-04	1.26E+01	1.10E+00	1.11E+00	2.79E-01	7.03E-02	1.21E-03	5.46E-01	2.14E-01	1.48E+01
acetic acid	2.50E-03	1.11E+02	7.92E+00	1.81E+00	1.44E-05	6.71E+01	1.22E-03	6.35E+01	3.66E+00	2.93E+00	5.88E-01	1.99E-01	1.97E-03	1.44E+00	7.03E-01	7.01E+01
dipropylene glycol monomethyl ether	5.64E-03	5.67E+02	1.79E+01	2.22E+00	7.71E-05	1.14E+02	4.05E-03	1.07E+02	6.30E+00	5.93E+00	1.27E+00	4.08E-01	3.76E-03	3.01E+00	1.24E+00	1.20E+02
alkylbenzene sulfonate	1.83E-03	6.87E+01	6.92E+00	3.09E-01	2.14E-05	5.84E+01	2.11E-03	5.75E+01	9.21E-01	1.30E+00	6.05E-01	4.02E-02	2.38E-03	4.69E-01	1.79E-01	5.97E+01
acrylic binder	2.48E-03	9.79E+01	9.42E+00	1.31E+00	2.25E-05	5.70E+01	3.05E-03	5.45E+01	2.51E+00	2.40E+00	6.87E-01	1.43E-01	2.46E-03	1.08E+00	4.82E-01	5.94E+01
brass	2.14E-02	3.80E+03	1.33E+02	5.06E+00	5.57E-03	7.89E+01	6.78E-03	6.87E+01	1.02E+01	1.90E+01	2.45E+00	4.97E-01	5.26E-03	1.41E+01	1.88E+00	9.79E+01
steel	5.13E-03	2.38E+02	2.60E+01	1.25E+00	1.26E-04	6.00E+01	3.82E-03	5.52E+01	4.77E+00	1.30E+01	2.15E+00	2.53E-01	3.38E-03	9.76E+00	8.71E-01	7.30E+01
wire drawing, steel	2.93E-04	1.40E+01	1.08E+00	6.68E-01	3.17E-06	3.22E+00	8.59E-03	2.99E+00	2.21E-01	4.22E-01	2.47E-01	1.56E-02	1.58E-04	1.15E-01	4.48E-02	3.64E+00
water, deionised	4.90E-07	1.61E+00	2.08E-03	8.64E-04	2.08E-08	6.25E-03	4.99E-07	5.71E-03	5.40E-04	5.45E-04	1.16E-04	3.17E-05	1.80E-05	2.78E-04	1.01E-04	6.80E-03

waste, from silicon wafer production	1.79E-04	8.21E+00	2.17E+00	2.78E-02	2.81E-07	1.57E+00	1.50E-03	1.53E+00	3.61E-02	2.97E-01	2.68E-01	2.04E-03	1.08E-04	1.96E-02	7.02E-03	1.87E+00
treatment, sewage	1.67E-03	2.75E+03	2.87E-02	5.17E-03	2.94E-08	5.19E-02	3.64E-06	5.05E-02	1.39E-03	3.20E-03	2.10E-03	7.43E-05	2.21E-06	7.71E-04	2.52E-04	5.51E-02
wafer factory	1.45E+02	9.96E+06	4.21E+06	3.35E+04	8.43E+00	1.25E+06	1.51E+02	1.16E+06	8.45E+04	2.21E+05	1.24E+05	3.99E+03	2.38E+03	7.61E+04	1.50E+04	1.47E+06
metallization paste, front side	1.33E+00	1.62E+05	4.65E+03	1.24E+02	6.60E-01	5.54E+03	7.56E-02	4.88E+03	6.64E+02	5.20E+02	9.06E+01	3.30E+01	1.53E-01	2.77E+02	1.20E+02	6.06E+03
metallization paste, back side	1.07E+00	1.31E+05	3.75E+03	1.00E+02	5.33E-01	4.49E+03	6.16E-02	3.95E+03	5.36E+02	4.20E+02	7.32E+01	2.66E+01	1.24E-01	2.23E+02	9.71E+01	4.91E+03
metallization paste, back side, aluminium	1.30E-02	4.04E+02	2.19E+01	2.97E+00	3.78E-05	1.31E+02	4.10E-03	1.27E+02	3.77E+00	1.19E+01	1.31E+00	1.02E-01	2.62E-03	1.01E+01	4.10E-01	1.43E+02
ammonia	1.68E-03	5.28E+01	6.46E+00	2.46E+00	9.60E-06	4.03E+01	3.27E-04	4.00E+01	3.01E-01	1.01E+00	7.22E-01	1.67E-02	9.98E-03	2.15E-01	4.75E-02	4.13E+01
phosphorus oxychloride	5.48E-03	1.62E+02	3.21E+01	1.74E+00	4.22E-05	6.87E+01	2.74E-02	5.96E+01	9.13E+00	7.53E+00	1.86E+00	4.73E-01	5.29E-02	3.41E+00	1.74E+00	7.63E+01
isopropanol	2.26E-03	1.17E+02	8.17E+00	7.43E-01	3.07E-05	7.46E+01	1.59E-03	7.32E+01	1.45E+00	1.60E+00	4.68E-01	8.88E-02	3.41E-03	7.51E-01	2.84E-01	7.62E+01
hydrochloric acid	9.51E-04	4.50E+01	3.34E+00	5.06E-01	2.75E-05	1.37E+01	7.36E-04	1.26E+01	1.10E+00	1.11E+00	2.79E-01	7.03E-02	1.21E-03	5.46E-01	2.14E-01	1.48E+01
hydrogen fluoride	2.35E-03	1.36E+02	1.04E+01	1.56E+00	1.85E-04	2.05E+01	2.99E-03	1.91E+01	1.42E+00	2.18E+00	7.69E-01	7.56E-02	1.11E-02	1.05E+00	2.66E-01	2.27E+01
sodium hydroxide	1.67E-03	5.83E+01	4.60E+00	7.05E-01	2.70E-05	1.95E+01	1.51E-03	1.73E+01	2.14E+00	1.90E+00	3.52E-01	1.38E-01	1.05E-03	9.89E-01	4.16E-01	2.13E+01
lime	3.57E-04	1.07E+01	2.53E+00	2.68E-02	3.62E-07	5.04E+00	9.31E-04	4.94E+00	1.03E-01	5.09E-01	2.74E-01	5.56E-03	4.66E-04	2.11E-01	1.85E-02	5.55E+00
refrigerant R134a	7.23E-03	2.23E+02	1.48E+01	3.68E+00	1.53E-04	1.15E+02	3.73E-03	1.07E+02	8.65E+00	5.26E+00	1.16E+00	2.84E-01	1.66E-02	2.63E+00	1.17E+00	1.21E+02
nitrogen	4.50E-04	1.22E+01	8.75E-01	4.36E-01	4.18E-07	5.77E+00	1.10E-04	5.11E+00	6.62E-01	5.79E-01	7.25E-02	4.85E-02	2.89E-05	3.24E-01	1.34E-01	6.35E+00

silane	2.02E-02	3.17E+02	1.18E+02	2.41E+01	3.60E-05	5.93E+02	3.80E-03	4.31E+02	1.62E+02	1.04E+02	4.14E+01	1.21E+00	2.34E+00	3.43E+01	2.46E+01	6.97E+02
tap water	1.35E-06	4.62E-02	3.16E-03	2.89E-04	6.06E-09	1.62E-02	5.27E-07	1.45E-02	1.75E-03	1.44E-03	2.12E-04	1.21E-04	7.98E-06	7.73E-04	3.30E-04	1.77E-02
photovoltaic cell factory	1.60E+03	7.17E+07	2.45E+07	3.64E+05	1.35E+01	1.41E+07	3.35E+03	1.35E+07	5.47E+05	7.86E+05	1.90E+05	2.91E+04	4.35E+03	4.58E+05	1.05E+05	1.49E+07
transport, freight lorry	4.51E-04	3.06E+00	1.64E+00	1.24E-02	6.43E-07	2.94E+00	1.05E-04	2.91E+00	3.53E-02	3.61E-02	9.58E-03	1.98E-03	3.33E-04	1.78E-02	6.44E-03	2.98E+00
transport, freight train	1.80E-04	1.15E+00	4.05E-01	6.54E-03	1.31E-07	6.89E-01	7.09E-05	6.58E-01	3.08E-02	2.97E-02	7.65E-03	1.57E-03	2.68E-05	1.49E-02	5.59E-03	7.19E-01
wastewater from PV cell production	9.26E-02	1.36E+02	5.77E+00	5.23E-01	4.71E-06	1.62E+01	5.86E-03	1.46E+01	1.59E+00	1.23E+00	1.97E-01	8.74E-02	7.22E-04	6.56E-01	2.87E-01	1.74E+01
waste, from silicon wafer production, inorganic	3.45E-04	1.33E+01	1.99E+00	2.20E-02	1.56E-06	2.23E+00	5.07E-04	2.17E+00	5.36E-02	9.16E-02	3.30E-02	4.12E-03	1.45E-04	4.22E-02	1.21E-02	2.32E+00
spent solvent mixture	4.48E-04	1.97E+01	5.82E-01	7.36E-02	1.62E-06	3.24E+00	1.41E-04	3.13E+00	1.17E-01	1.15E-01	2.63E-02	7.48E-03	5.01E-04	5.85E-02	2.27E-02	3.36E+00
aluminium alloy, AlMg3	8.08E-03	2.82E+02	1.74E+01	1.81E+00	8.10E-05	8.35E+01	4.18E-03	7.76E+01	5.88E+00	8.84E+00	1.20E+00	2.31E-01	4.94E-02	6.37E+00	9.82E-01	9.24E+01
copper	6.78E-03	1.36E+03	6.50E+01	1.19E+00	3.08E-03	1.59E+01	1.23E-03	1.43E+01	1.63E+00	4.17E+00	4.54E-01	1.09E-01	1.55E-03	3.27E+00	3.31E-01	2.01E+01
wire drawing, copper	1.48E-03	2.21E+02	8.46E+00	4.89E-01	2.87E-04	9.73E+00	3.77E-04	8.34E+00	1.40E+00	1.67E+00	3.18E-01	6.55E-02	4.76E-04	1.03E+00	2.57E-01	1.14E+01
diode	3.02E-01	7.48E+03	6.24E+02	8.82E+01	3.75E-04	3.53E+03	3.26E-02	3.05E+03	4.81E+02	3.76E+02	7.26E+01	2.60E+01	9.41E-02	1.90E+02	8.73E+01	3.91E+03
silicone product	3.52E-03	1.51E+02	1.56E+01	4.93E+00	2.57E-05	5.90E+01	2.79E-03	5.54E+01	3.59E+00	5.17E+00	2.29E+00	1.70E-01	1.43E-03	2.12E+00	5.86E-01	6.42E+01
tin	2.97E-02	3.85E+03	8.95E+01	7.59E+00	2.69E-02	1.42E+02	5.09E-03	1.27E+02	1.49E+01	1.89E+01	7.70E+00	8.05E-01	4.38E-03	7.59E+00	2.76E+00	1.61E+02
lead	2.11E-03	3.23E+02	1.35E+01	1.12E+00	1.32E-03	1.77E+01	1.86E-03	1.60E+01	1.69E+00	2.15E+00	5.86E-01	1.17E-01	1.27E-03	1.07E+00	3.81E-01	1.99E+01

solar glass, low-iron	1.82E-03	2.61E+01	4.05E+00	2.40E-01	9.30E-06	1.31E+01	1.51E-03	1.27E+01	4.02E-01	5.66E-01	3.03E-01	1.81E-02	9.85E-04	1.71E-01	7.36E-02	1.37E+01
tempering, flat glass	2.15E-04	3.31E+00	4.10E-01	3.07E-02	1.10E-06	2.37E+00	4.25E-05	2.33E+00	4.87E-02	6.80E-02	3.62E-02	2.13E-03	1.50E-04	2.06E-02	8.91E-03	2.44E+00
glass fibre reinforced plastic	1.22E-02	5.08E+01	1.04E+01	8.87E+00	2.11E-05	1.45E+02	2.85E-03	1.29E+02	1.57E+01	4.53E+00	2.15E+00	1.56E-01	2.34E-03	1.48E+00	7.37E-01	1.49E+02
polyethylene terephthalate	3.04E-03	1.10E+02	1.17E+01	1.03E+00	3.50E-05	8.56E+01	4.02E-03	8.22E+01	3.34E+00	3.02E+00	7.74E-01	1.59E-01	1.21E-02	1.45E+00	6.18E-01	8.86E+01
polyethylene, high density	2.21E-03	9.68E+01	8.80E+00	7.81E-01	2.23E-05	8.71E+01	2.27E-03	8.51E+01	2.01E+00	1.82E+00	4.85E-01	1.06E-01	4.50E-03	8.50E-01	3.80E-01	8.89E+01
ethylvinylacetate	3.13E-03	1.18E+02	1.86E+01	1.85E+00	2.62E-05	9.92E+01	3.80E-03	9.47E+01	4.59E+00	4.57E+00	1.95E+00	2.05E-01	3.82E-03	1.56E+00	8.46E-01	1.04E+02
polyvinylfluoride	2.00E-02	8.88E+02	4.37E+01	6.94E+00	1.73E-04	2.44E+02	6.57E-03	2.20E+02	2.43E+01	1.84E+01	3.53E+00	1.28E+00	2.00E-02	9.26E+00	4.31E+00	2.63E+02
tap water	1.35E-06	4.62E-02	3.16E-03	2.89E-04	6.06E-09	1.62E-02	5.27E-07	1.45E-02	1.75E-03	1.44E-03	2.12E-04	1.21E-04	7.98E-06	7.73E-04	3.30E-04	1.77E-02
hydrogen fluoride	2.35E-03	1.36E+02	1.04E+01	1.56E+00	1.85E-04	2.05E+01	2.99E-03	1.91E+01	1.42E+00	2.18E+00	7.69E-01	7.56E-02	1.11E-02	1.05E+00	2.66E-01	2.27E+01
1-propanol	7.47E-03	2.97E+02	2.37E+01	3.50E+00	3.16E-05	1.37E+02	2.54E-03	1.31E+02	5.39E+00	5.45E+00	2.01E+00	2.84E-01	4.50E-03	2.15E+00	9.98E-01	1.42E+02
isopropanol	2.26E-03	1.17E+02	8.17E+00	7.43E-01	3.07E-05	7.46E+01	1.59E-03	7.32E+01	1.45E+00	1.60E+00	4.68E-01	8.88E-02	3.41E-03	7.51E-01	2.84E-01	7.62E+01
potassium hydroxide	5.12E-03	1.54E+03	1.94E+01	1.28E+00	8.83E-05	6.37E+01	4.80E-03	5.65E+01	7.15E+00	6.22E+00	1.41E+00	4.00E-01	6.93E-03	2.94E+00	1.47E+00	6.99E+01
soap	2.31E-02	5.07E+02	1.68E+02	8.78E+00	2.02E-05	2.32E+01	3.26E+00	1.84E+01	1.54E+00	8.24E+01	8.13E+01	1.11E-01	9.13E-03	6.64E-01	2.95E-01	1.06E+02
corrugated board box	2.93E-03	1.21E+02	7.57E+01	5.27E-01	4.41E-06	1.74E+01	2.97E-02	1.63E+01	1.10E+00	1.40E+01	1.34E+01	5.69E-02	1.02E-03	3.74E-01	1.74E-01	3.14E+01
EUR-flat pallet	1.59E-02	3.29E+02	4.59E+03	4.07E+00	3.82E-05	1.30E+02	5.57E-02	1.25E+02	5.30E+00	6.28E+02	6.24E+02	3.38E-01	1.61E-02	3.19E+00	1.06E+00	7.58E+02
photovoltaic panel factory	4.99E+02	1.56E+07	1.74E+07	8.76E+04	8.72E+00	3.75E+06	6.32E+02	3.56E+06	1.90E+05	9.52E+05	7.84E+05	8.35E+03	1.46E+03	1.26E+05	3.21E+04	4.70E+06

municipal solid waste	1.64E-04	3.07E+00	2.23E-01	6.85E-02	1.06E-07	3.52E-01	5.45E-04	3.44E-01	7.69E-03	1.06E-02	3.53E-03	4.16E-04	2.33E-05	5.14E-03	1.50E-03	3.63E-01
waste polyvinylfluoride	3.22E-04	7.25E+00	1.16E+00	3.18E-01	9.32E-07	1.69E+00	3.88E-04	1.63E+00	6.01E-02	9.00E-02	3.12E-02	3.55E-03	1.38E-04	4.29E-02	1.22E-02	1.78E+00
waste plastic, mixture	3.09E-04	5.64E+00	1.31E-01	1.57E-01	1.71E-07	5.05E-01	3.80E-05	4.92E-01	1.36E-02	1.85E-02	6.28E-03	7.22E-04	5.73E-05	8.82E-03	2.65E-03	5.24E-01
waste mineral oil	9.24E-05	1.46E+00	1.02E-01	3.56E-02	2.06E-07	3.33E-01	2.65E-05	3.23E-01	9.71E-03	1.30E-02	4.69E-03	5.66E-04	3.21E-05	5.80E-03	1.88E-03	3.46E-01
treatment, sewage	1.67E-03	2.75E+03	2.87E-02	5.17E-03	2.94E-08	5.19E-02	3.64E-06	5.05E-02	1.39E-03	3.20E-03	2.10E-03	7.43E-05	2.21E-06	7.71E-04	2.52E-04	5.51E-02
**Recycling of 1m² Silicon PV Module**																
electricity, medium voltage (ENTSO-E)	2.62E-04	7.93E+00	1.34E+00	2.04E-01	7.21E-07	1.87E+00	7.62E+00	4.42E-05	4.00E+00	3.62E+00	2.69E-01	1.15E-01	4.11E-05	7.93E-01	6.92E-01	9.49E+00
diesel	4.19E-04	8.47E-01	9.22E-02	3.21E-03	3.68E-08	8.04E-03	1.39E+00	1.08E-05	1.38E+00	9.24E-03	1.53E-03	4.45E-04	4.05E-05	4.37E-03	1.66E-03	1.40E+00
waste plastic plaster	2.77E-05	7.44E-01	5.71E-01	1.81E-03	2.39E-08	6.87E-03	2.53E-01	3.00E-06	2.47E-01	5.94E-03	9.84E-04	1.05E-04	1.18E-05	5.23E-03	5.45E-04	2.60E-01
natural gas	6.35E-05	5.32E-01	1.76E-02	1.25E-03	3.01E-08	2.48E-03	1.30E+00	5.78E-06	1.30E+00	2.97E-03	5.74E-04	1.65E-04	2.94E-06	1.29E-03	4.50E-04	1.30E+00
heavy fuel oil	6.14E-05	3.98E-01	5.97E-02	1.28E-03	9.46E-09	4.27E-03	1.23E+00	3.22E-06	1.22E+00	6.83E-03	6.63E-04	2.19E-04	2.02E-06	2.20E-03	1.19E-03	1.23E+00
silica sand	4.49E-05	6.61E-01	9.72E-01	3.96E-03	5.73E-08	2.88E-02	3.38E-01	9.04E-06	3.29E-01	9.06E-03	2.33E-02	4.00E-04	7.05E-05	3.48E-03	1.51E-03	3.66E-01
soda, powder	4.64E-04	5.15E+01	3.69E+00	7.28E-01	1.90E-05	7.73E-01	4.20E+00	1.37E-04	3.79E+00	4.14E-01	5.36E-01	1.44E-02	3.66E-04	1.51E-01	7.17E-02	4.97E+00
limestone	1.84E-05	2.23E+00	1.60E-02	3.64E-03	6.80E-09	3.49E-03	3.15E-02	7.46E-07	3.04E-02	1.03E-03	1.39E-03	1.49E-05	1.51E-05	1.99E-03	8.17E-05	3.49E-02
aluminium	2.42E-02	7.28E+02	3.90E+01	3.14E+00	1.59E-05	1.26E+01	2.19E+02	2.60E-03	2.16E+02	2.60E+00	2.22E+00	9.97E-02	1.87E-03	9.77E+00	4.63E-01	2.31E+02

Supplementary references:

1. Stolz, P., Frischknecht, R., Wambach, K., Sinha, P. & Heath, G. Life cycle assessment of current photovoltaic module recycling. *IEA PVPS Task 12, International Energy Agency Power Systems Programme, Report IEA-PVPS T12* **13**, 2018 (2017).
2. Nafeesah Allen, C. T., Samantha Allen. Solar Panel Size And Weight: A Comprehensive Guide. https://www.forbes.com/home-improvement/solar/solar-panel-size-weight-guide/ (2024).
3. Chen, B. *et al.* Recycling lead and transparent conductors from perovskite solar modules. *Nat. Commun.* **12**, 5859 (2021).
4. Park, S. Y. *et al.* Sustainable lead management in halide perovskite solar cells. *Nat. Sustain.* **3**, 1044-1051 (2020).
5. Abundance in Earth Crust. https://www.science.co.il/elements/.
6. Wagner, L. *et al.* The resource demands of multi-terawatt-scale perovskite tandem photovoltaics. *Joule* **8**, 1142-1160 (2024).
7. Vidal, R., Alberola-Borràs, J.-A. & Mora-Seró, I. Abiotic depletion and the potential risk to the supply of cesium. *Resour. Policy* **68**, 101792 (2020).
8. Binek, A. *et al.* Recycling perovskite solar cells to avoid lead waste. *ACS Appl. Mater. Interfaces* **8**, 12881-12886 (2016).
9. Wang, K. *et al.* "One-key-reset" recycling of whole perovskite solar cell. *Matter* **4**, 2522-2541 (2021).
10. Liu, F.-W. *et al.* Recycling and recovery of perovskite solar cells. *Mater. Today* **43**, 185-197 (2021).
11. Capello, C., Fischer, U. & Hungerbühler, K. What is a green solvent? A comprehensive framework for the environmental assessment of solvents. *Green Chem.* **9**, 927-934 (2007).

12 Kadro, J. M. *et al.* Proof-of-concept for facile perovskite solar cell recycling. *Energy Environ. Sci.* **9**, 3172-3179 (2016).

13 Prat, D. *et al.* CHEM21 selection guide of classical-and less classical-solvents. *Green Chem.* **18**, 288-296 (2016).

14 Byrne, F. P. *et al.* Tools and techniques for solvent selection: green solvent selection guides. *Sustain. Chem. Process.* **4**, 1-24 (2016).

15 NIOSH. Preventing Adverse Health Effects from Exposure to: Dimethylformamide (DMF). https://www.cdc.gov/niosh/docs/90-105.

16 Roose, P., Turcotte, M. G. & Mitchell, J. W. Methylamines. *Kirk-Othmer Encyclopedia of Chemical Technology*, 1-16 (2000).

17 Doolin, A. J. *et al.* Sustainable solvent selection for the manufacture of methylammonium lead triiodide ($MAPbI_3$) perovskite solar cells. *Green Chem.* **23**, 2471-2486 (2021).

18 Podapangi, S. K. *et al.* Green solvents, materials, and lead-free semiconductors for sustainable fabrication of perovskite solar cells. *RSC Adv.* **13**, 18165-18206 (2023).

19 Tian, X., Stranks, S. D. & You, F. Life cycle assessment of recycling strategies for perovskite photovoltaic modules. *Nat. Sustain.* **4**, 821-829 (2021).

20 Li, C. *et al.* Rational design of Lewis base molecules for stable and efficient inverted perovskite solar cells. *Science* **379**, 690-694 (2023).

21 Shargaieva, O., Kuske, L., Rappich, J., Unger, E. & Nickel, N. H. Building blocks of hybrid perovskites: A photoluminescence study of lead-iodide solution species. *ChemPhysChem* **21**, 2327-2333 (2020).

22 Ecoinvent Version 3.10. *Ecoinvent Centre* (https://www.ecoinvent.org/, Jan 1st, 2024).

23 Bi, D. *et al.* Efficient luminescent solar cells based on tailored mixed-cation perovskites. *Sci. Adv.* **2**, e1501170 (2016).

24 Gong, J., Darling, S. B. & You, F. Perovskite photovoltaics: life-cycle assessment of energy and environmental impacts. *Energy Environ. Sci.* **8**, 1953-1968 (2015).

25 Ahangharnejhad, R. H. *et al.* Impact of lifetime on the levelized cost of electricity from perovskite single junction and tandem solar cells. *Sustain. Energy Fuel.* **6**, 2718-2726 (2022).

26 Song, Z. *et al.* A technoeconomic analysis of perovskite solar module manufacturing with low-cost materials and techniques. *Energy Environ. Sci.***10**, 1297-1305 (2017).

27 Li, Z. *et al.* Cost analysis of perovskite tandem photovoltaics. *Joule* **2**, 1559-1572 (2018).
